# Real-time computation of brain E-field for enhanced transcranial magnetic stimulation neuronavigation and optimization

**DOI:** 10.1162/imag_a_00412

**Published:** 2025-01-02

**Authors:** Nahian I. Hasan, Moritz Dannhauer, Dezhi Wang, Zhi-De Deng, Luis J. Gomez

**Affiliations:** Elmore Family School of Electrical and Computer Engineering, Purdue University, West Lafayette, IN, United States; Computational Neurostimulation Research Program, Noninvasive Neuromodulation Unit, Experimental Therapeutics and Pathophysiology Branch, National Institute of Mental Health Intramural Research Program, National Institutes of Health, Bethesda, MD, United States; Department of Computer Science, Center for Brain Stimulation, East Carolina University, Greenville, NC, United States

**Keywords:** real-time, transcranial magnetic stimulation, Huygens’ principle, PMD, FEM, neuronavigation

## Abstract

Transcranial Magnetic Stimulation (TMS) coil placement and pulse waveform current are often chosen to achieve a specified E-field dose on targeted brain regions. TMS neuronavigation could be improved by including real-time accurate distributions of the E-field dose on the cortex. We introduce a method and develop software for computing brain E-field distributions in real-time enabling easy integration into neuronavigation and with the same accuracy as$1^{\text{st}}$-order finite element method (FEM) solvers. Initially, a spanning basis set (<400) of E-fields generated by white noise magnetic currents on a surface separating the head and permissible coil placements are orthogonalized to generate the modes. Subsequently, Reciprocity and Huygens’ principles are utilized to compute fields induced by the modes on a surface separating the head and coil by FEM, which are used in conjunction with online (real-time) computed primary fields on the separating surface to evaluate the mode expansion. We conducted a comparative analysis of E-fields computed by FEM and in real-time for eight subjects, utilizing two head model types (SimNIBS’s ‘headreco’ and ‘mri2mesh’ pipeline), three coil types (circular, double-cone, and Figure-8), and 1000 coil placements (48,000 simulations). The real-time computation for any coil placement is within 4 milliseconds (ms), for 400 modes, and requires less than 4 GB of memory on a GPU. Our solver is capable of computing E-fields within 4 ms, making it a practical approach for integrating E-field information into the neuronavigation systems without imposing a significant overhead on frame generation. The software is available athttps://github.com/NahianHasan/Real-Time-TMS.

## 1 Introduction

Transcranial magnetic stimulation (TMS) (Barker et al., 1985;Paulus et al., 2013) is a non-invasive brain stimulation approach that is widely used in neuroscience research to study brain function and has been approved by the Food and Drug Administration in the treatment of depression, obsessive-compulsive disorder, migraine, and smoking (Carmi et al., 2019;Cohen et al., 2022;Couturier, 2005;De Ridder et al., 2007;Hoffman et al., 2005;Kaptsan et al., 2003;Lan et al., 2017;Lipton et al., 2010;Nahas et al., 2003;O’Reardon et al., 2007;Pascual-Leone et al., 1996). TMS uses coils driven by low-frequency current pulses to stimulate targeted brain regions. Computational electric field (E-field) dosimetry can be used to 1) quantify the E-field strength and distribution to determine brain regions affected by TMS, 2) identify coil placements and orientation that would maximize the E-field induced at a prespecified target, and 3) design coils with a desired E-field profile (Beynel et al., 2020;Dannhauer et al., 2022;Gomez et al., 2018,^2021^;Laakso & Hirata, 2012;D. Wang et al., 2023;Weise et al., 2023). All of these applications require repeated execution of E-field solvers to determine the E-field. Correspondingly, there is ongoing interest in fast and accurate E-field solvers for TMS (Daneshzand et al., 2021;Gomez et al., 2021;Stenroos & Koponen, 2019). In particular, there is an interest in incorporating accurate and precise E-field information in neuronavigation systems that use subject Magnetic Resonance Imaging (MRI) along with cameras to provide practitioners with precise information about the coil location relative to the head. Appending E-field information to neuronavigation tools would allow for on-the-fly E-field informed coil placement reconfiguration and intensity dosing to target multiple or even changing cortical targets during a single TMS session (Daneshzand et al., 2023;Nieminen et al., 2022). The integration of E-field information into neuronavigation requires a solver that can accurately determine the E-field in real-time.

Several approaches have been proposed for determining the E-field in real-time. For example, the most widely used approach locally fits a sphere to the head to estimate the E-field under the coil rapidly (Nummenmaa et al., 2013;Sollmann et al., 2016). However, this solver does not account for gyral folds of the cerebrospinal fluid and gray matter boundary surface, consequently, yielding less accurate local E-field estimates (Nummenmaa et al., 2013). Furthermore, deep learning approaches have been introduced, which have been promising. However, at this point, they still result in a high relative residual error of about 6% even in a small target region (Yokota et al., 2019) and 18% in the whole brain in (Xu et al., 2021). More recently, boundary element method (BEM)-based near real-time solvers that can account for local brain anatomy have been developed (Daneshzand et al., 2021;Stenroos & Koponen, 2019).

The elegant method of (Stenroos & Koponen, 2019) uses efficient quadratures for the coil and tissue boundary sources enabling real-time E-field solutions using GPUs. For example, the authors developed coil dipole models that achieved the same accuracy as those with thousands of dipoles but with only 42 dipoles per layer. Additionally, they created BEM meshes that properly accounted for gyral geometry while only requiring 21,052 nodes. These efficient quadratures coupled with a highly optimized hardware implementation of BEM enabled the approximation of the E-field in real time. Specifically, they use pre-computed boundary potentials on a mesh to compute the TMS-induced E-field in a cortical region of interest (ROI) in real-time via reciprocity. Using their method, the computation scales as the product of ‘the number of cortical E-field evaluation points’ times ‘the number of vertices of the surface meshes’ plus ‘the number of coil quadrature nodes’. As a result, a trade-off between the number of mesh nodes and the number of cortical evaluation points must be made to keep the computation low. These computations are done rapidly by leveraging massively parallel architectures like GPUs, enabling the mitigation of asymptotic computational costs for many practical scenarios.

To maintain fast computation, there must be a trade-off between mesh resolution and the number of ROI points. For example, to achieve 36 ms on a GPU they used a mesh consisting of 21,052 nodes and 20,324 cortical ROI locations. Recently, the authors introduced an improved GPU-accelerated implementation of the method (Stenroos et al., 2023), where it took 20.41 ms for a 42 dipole coil model and 22.73 ms with a 15,000 dipole coil model to compute the E-field for a single coil placement over the same head model. The Ernie mesh, a typical head mesh generated by SimNIBS software v3.2 (Thielscher et al., 2015), has more than 10 times the number of cortical surface nodes (216,130 nodes). Such a computation cannot be achieved using current hardware in real-time via the above method. Furthermore, newer more detailed head models generated by using SimNIBS v4.0 contain an even higher number of nodes. As a result, we aim to extend real-time solvers to analyze E-fields while using these denser and more detailed meshes that are typically used in the non-invasive brain stimulation community for modeling.

To overcome these challenges, Daneshzand et al. proposed the Magnetic Stimulation Profile (MSP) approach that approximates the TMS coil-induced E-fields in a cortical ROI as a linear combination of dipole-induced E-fields (Daneshzand et al., 2021). Dipole-induced E-fields are precomputed in a process that can take 5 to 18 hours depending on desired accuracy and mesh resolution. Then, these precomputed E-fields are used in real-time to determine the TMS coil-induced E-fields. To find E-field expansion coefficients, the method employs a least squares to match the primary E-field of the coil and the primary E-fields induced by a linear combination of dipoles. This method requires∼3000 dipole E-fields, requiring∼32 GBsof memory and about 0.37 s using a Xeon E5-2360 CPU for 120,000 cortical triangles to match the full BEM solution with an average relative residual error of about 10% and a 5% error in predicting the peak E-field (Daneshzand et al., 2021). The memory and CPU time of this method scales as the number of dipole E-fields times the number of E-field evaluation points. As such, to lower the memory requirements and achieve real-time performance, trade-offs between accuracy and cortical E-field samples must be made.

In this paper, similar to the MSP approach, we approximate the E-field as a linear combination of precomputed basis E-fields. However, we use a novel method for determining an E-field basis instead of the dipole E-field basis. This results in a more accurate E-field solution for a fixed number of precomputed basis E-fields. To find an efficient basis of E-field modes on the cortex due to any source outside of the head, we apply an approach similar to the probabilistic matrix decomposition (PMD) based approach (Hasan et al., 2023). By using modes instead of dipole E-fields, we reduce the required number of modes more than 10-fold relative to the MSP approach. For example, to reach a 10% error, we require 110 modes relative to the 3000 modes required using the MSP approach. Furthermore, our approach requires under 400 basis modes to estimate the TMS-induced E-field with an error lower than 3%. Additionally, we introduce a novel method to determine the expansion coefficients from the primary E-fields and magnetic field (H-field) on a fictitious surface engulfing the head that minimizes the energy of the error of the predicted field. Using this method, we can estimate the E-field to 2% error within 4 ms from the primary E-field and H-field for 216,000 cortical surface targets. Finally, unlike the MSP, the expansion coefficients are found analytically using reciprocity and Huygens’s principle formalism to determine the coefficients without the need for numerical techniques requiring regularization to prevent over-fitting.

## 2 Methods

### 2.1 Overview

The notations used in this article are summarized inAppendix Table 1. This section describes the procedure for real-time determination of an approximate expansion for the E-field induced by the coil in the brain during TMS

$$\begin{array}{l} {\text{E}}_{\text{TMS}}\left(\text{r};t\right)\approx {\text{E}}_{\text{TMS}}^{\left(N_{m}\right)}(\text{r};t)={\text{I}}^{\prime }\left(t\right){\text{E}}_{\text{TMS}}^{\left(N_{m}\right)}\left(\text{r}\right)={\text{I}}^{\prime }\left(t\right)\sum_{i=1}^{N_{m}}a^{\left(i\right)}{\text{M}}^{\left(i\right)}\left(\text{r}\right),\\ \;\;\;\text{r}\in \Omega . \end{array}$$ (1)

Here,rdenotes a Cartesian location,Ωdenotes the brain region,${\text{M}}^{\left(i\right)}\left(\text{r};t\right)=I^{\prime }\left(t\right){\text{M}}^{\left(i\right)}\left(\text{r}\right)$and$a^{\left(i\right)}$are one of the$N_{m}$mode functions and expansion coefficients, respectively,${\text{I}}^{\prime }\left(t\right)$is time-derivative of the driving current pulse waveform. Here, we assume that${\text{I}}^{\prime }\left(t\right)$is normalized to have a maximum time derivative of one. Correspondingly, this results in${\text{E}}_{\text{TMS}}^{\left(N_{m}\right)}\left(\text{r}\right)$being the peak E-field observed. Note that above and in the rest of this paper, we assume the TMS-induced E-fields are quasi-static and this is standard for TMS E-field modeling (Daneshzand et al., 2021;Gomez et al., 2020b,^2021^;Plonsey, 1972;Thielscher et al., 2015;D. Wang et al., 2023;B. Wang et al., 2024). As a result, we assume that the TMS coil driving current is separable${\text{J}}_{\text{TMS}}\left(\text{r};t\right)=\text{I}\left(t\right){\text{J}}_{\text{TMS}}\left(\text{r}\right)$and the H-fields and E-fields are proportional to the coil driving current and its temporal derivative, respectively (more details can be found in section 6.1 and 6.2 of theSupplementary File).

InSection 2.2, we describe a procedure for finding the spatial variation of the orthonormal mode functions${\text{M}}^{\left(i\right)}\left(\text{r}\right)$(i.e.,$\langle {\text{M}}^{\left(i\right)},{\text{M}}^{\left(j\right)}\rangle =\delta_{i,j}$, where$\langle \text{f},\text{g}\rangle =\int_{\Omega }\text{f}\left(\text{r}\right)\cdot \text{g}\left(\text{r}\right)\text{dr}$and$\delta_{i,j}$is the Kronecker delta function) that can efficiently represent the E-fields induced in the brain by any TMS coil. Once these functions are determined, the coefficients$a^{\left(i\right)}$are chosen to minimize the$L^{2}$error of the expansion (i.e.,${\text{argmin}}_{a\in {\mathbb{R}}^{N_{m}}}\left\|\;E_{\mathrm{TMS}}-{\text{E}}_{\text{TMS}}^{\left(N_{m}\right)}\;\right\|$, where${\text{E}}_{\text{TMS}}$is the E-field modeled using the in-house built field solver,$\left\|\;\text{f}\;\right\|=\sqrt{\;\left\|\;\text{f},\text{f}\;\right\|}$, and$\text{a}=\left(a^{\left(1\right)},a^{\left(2\right)},...,a^{\left(N_{m}\right)}\right))$. Since the mode functions are orthonormal, the coefficients are$a^{\left(i\right)}=\langle {\text{M}}^{\left(i\right)},\;{\text{E}}_{\text{TMS}}\rangle$. The above expression for determining each$a^{\left(i\right)}$requires*a priori*knowledge of the TMS-induced E-field in the brain. As such, it is not amenable to real-time solvers. Alternatively, inSection 2.3, we use reciprocity and Huygens’s equivalence principles (Balanis, 2012) to show that

$$a^{\left(i\right)}=\int S\left[{\text{E}}_{\text{TMS}}^{\text{P}}\left(\text{r}\right)\cdot {\text{J}}_{\text{S}}^{\left(i\right)}\left(\text{r}\right)-{\text{H}}_{\text{TMS}}^{\text{P}}\left(\text{r}\right)\cdot {\text{K}}_{\text{S}}^{\left(i\right)}\left(\text{r}\right)\right]\text{dr}.$$ (2)

Here,Sdenotes the Huygens’s surface separating the head and the coil,${\text{J}}_{\text{S}}^{\left(i\right)}\left(\text{r};t\right)=\text{I}\left(t\right){\text{J}}_{\text{S}}^{\left(i\right)}\left(\text{r}\right)$and${\text{K}}_{\text{S}}^{\left(i\right)}\left(r;t\right)={\text{I}}^{\prime }\left(t\right){\text{K}}_{\text{S}}^{\left(i\right)}\left(\text{r}\right)$are fictitious equivalent electric and magnetic current densities associated with the$i^{\text{th}}$mode function, respectively, and${\text{E}}_{\text{TMS}}^{\text{P}}\left(\text{r};t\right)={\text{I}}^{\prime }\left(t\right){\text{E}}_{\text{TMS}}^{\text{P}}\left(\text{r}\right)$and${\text{H}}_{\text{TMS}}^{\text{P}}\left(\text{r};t\right)=\text{I}\left(t\right){\text{H}}_{\text{TMS}}^{\text{P}}\left(\text{r}\right)$are the primary E-field and H-field, respectively.Equation (2)only requires primary fields on Huygens’s surface and here it is used to determine expansion coefficients in real-time. Insection 2.5, we provide a method for rapidly determining${\text{E}}_{\text{TMS}}^{\text{P}}\left(\text{r}\right)$and${\text{H}}_{\text{TMS}}^{\text{P}}\left(r\right)$on the Huygens’s surface.Section 2.6summarizes the pre-processing and real-time stages for an easy implementation. Finally,sections 2.7and2.8describe the head and coil models for validating the algorithm and error quantization metrics, respectively.

### 2.2 Generation of mode function ${\text{M}}^{\left(i\right)}$

**Fig. 1.**
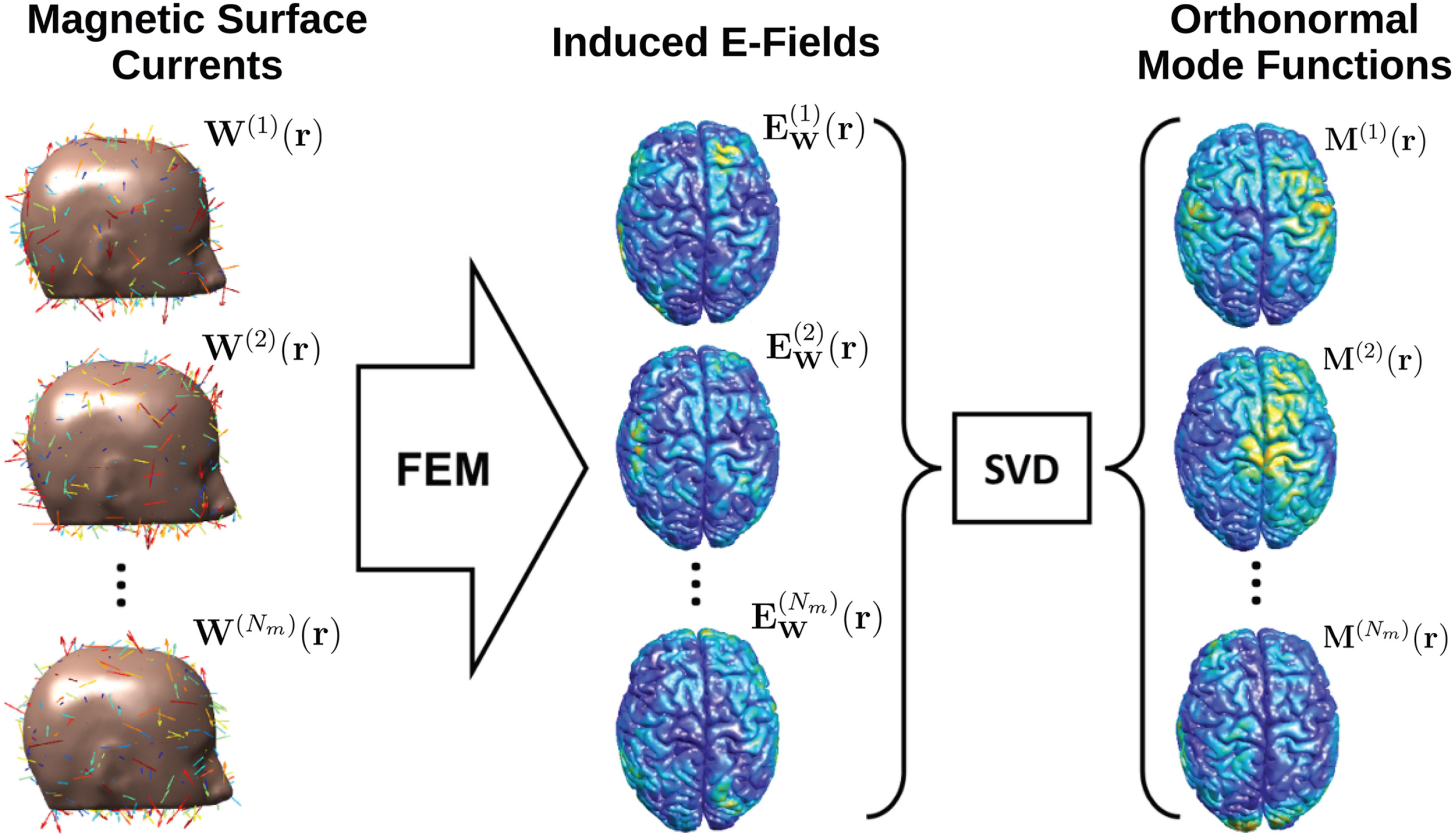
Generation of Mode Functions from surface magnetic currents. The left column shows the individual realization of magnetic surface currents$\left(W^{\left(i\right)}\left(\text{r}\right)\right)$. The middle column shows the induced E-field on the brain for each surface current distribution, generated by an FEM simulation. The right column shows the$N_{m}$orthonormal mode functions [${\text{M}}^{\left(i\right)}\left(\text{r}\right)$,$i=1,2,...,N_{m}$], generated by a singular value decomposition (SVD) over the$N_{m}$induced E-fields.

The mode functions${\text{M}}^{\left(i\right)}\left(\text{r};t\right)$, where$i=1,2,...,N_{m}$, are chosen as an orthonormal basis to E-fields induced in the brain by$N_{m}$magnetic surface current density distributions residing on a fictitious surface 1 mm directly outside of the scalp (Fig. 1). The reason we chose magnetic surface current distributions is that they do not need to be divergence-free, in fact, they are oriented randomly on the fictitious surface at each point in space with a normal distribution. The$N_{m}$current density distributions are chosen as distinct realizations of Gaussian white noise${\text{W}}^{\left(i\right)}\left(\text{r};t\right)=I^{\prime }\left(t\right){\text{W}}^{\left(i\right)}\left(\text{r}\right)$. The$N_{m}$Gaussian white noise magnetic current density realizations are continuous analogous to the Gaussian white noise vectors that we previously successfully used to compress matrices of TMS-induced brain E-field samples for many coil placements and brain locations (Hasan et al., 2023).

To generate each Gaussian white noise distributed current density, we first create a triangle mesh by extruding the nodes of the scalp surface mesh 1 mm normally outward. Then, we approximate the current as a piece-wise constant within each of the$N_{d}$triangles of the triangle mesh. The value of the current density is determined using a Gaussian distributed random number generator. To determine the primary (or free space) E-field${\text{E}}_{\text{W}}^{\text{P}\left(i\right)}\left(\text{r};t\right)={\text{I}}^{\prime }\left(t\right){\text{E}}_{\text{W}}^{\text{P}\left(i\right)}\left(\text{r}\right)$for the$i^{\text{th}}$surface current realization, we apply a single point quadrature to obtain

$$\begin{array}{l} {\text{E}}_{\text{W}}^{\text{P}\left(i\right)}\left(\text{r}\right)=-\frac{1}{4\pi }\int S{\text{W}}^{\left(i\right)}\left({\text{r}}^{\prime }\right)\times \nabla^{\prime }\frac{1}{\left|\text{r}-{\text{r}}^{\prime }\right|}{\text{dr}}^{\prime }\\ \;\;\;\;\;\;\;\;\;\;\;\;\approx -\sum_{j=1}^{N_{d}}A_{j}\frac{{\text{W}}^{\left(i\right)}\left({{\text{r}}^{\prime }}_{j}\right)\times \left(r-{{\text{r}}^{\prime }}_{j}\right)}{4\pi |\text{r}-{{\text{r}}^{\prime }}_{j}{|}^{3}}, \end{array}$$ (3)

where$A_{j}$and${\text{W}}^{\left(i\right)}({{\text{r}}^{\prime }}_{j})$are the area and the$i^{\text{th}}$realization of magnetic surface current at the center of the$j^{\text{th}}$triangle, respectively. The value of${\text{W}}^{\left(i\right)}({{\text{r}}^{\prime }}_{j})$is determined using a normally distributed random number generator.$\left\{{\text{W}}^{\left(i\right)}\left(\text{r};t\right);r\in S\right\}$can be simply considered as$i^{\text{th}}$realization of random magnetic dipoles on the surfaceS.

The presence of the head will distort the primary E-field. As a result, the total E-field induced in the head by current${\text{W}}^{\left(i\right)}\left(\text{r};t\right)$is${\text{E}}_{\text{W}}^{\left(i\right)}\left(\text{r};t\right)={\text{I}}^{\prime }\left(t\right)\left\{{\text{E}}_{\text{W}}^{\text{P}\left(i\right)}\left(\text{r}\right)-\nabla \phi^{\left(i\right)}\left(\text{r}\right)\right\}$, where$\phi^{\left(i\right)}\left(\text{r}\right)$is the scalar potential. Applying the current continuity to the conduction current results in the Poisson’sEquation (4)that can be solved for scalar potential-

$$\nabla \cdot \sigma \left(\text{r}\right)\nabla \phi^{\left(i\right)}\left(\text{r}\right)=\nabla \cdot \sigma \left(\text{r}\right){\text{E}}_{\text{W}}^{\text{P}\left(i\right)}\left(\text{r}\right);\text{r}\in \mathrm{Head},$$ (4)

whereσ(r)is the conductivity at Cartesian locationrin the head and the temporal variations are canceled from both sides. The region surrounding the head is assumed to be insulating (i.e.,σ(r)=0). The mesh is truncated on the outermost boundary of the head (i.e., the scalp boundary) where the normal component of the current density is continuous across the boundary. In other words,$\hat{\text{n}}\cdot \nabla \phi^{\left(i\right)}\left(\text{r}\right)=\hat{\text{n}}\cdot {\text{E}}_{\text{W}}^{\text{P}\left(i\right)}\left(\text{r}\right)$on the scalp boundary. Here,$\hat{\text{n}}$is a normal vector on the scalp surface pointing outward. To solveEquation (4), we approximate the head by a tetrahedral mesh where each tetrahedron is assigned a homogeneous conductivity.

Then,Equation (4)is solved using either our$1^{\text{st}}$- or 2^nd^-order in-house finite element solvers that have been cross-verified (Gomez et al., 2020b) and available online (Gomez et al., 2020a).

The spatial variation of the total E-field in the brain is approximated as a piece-wise constant within each tetrahedron. The resulting expansion is${\text{E}}_{\text{W}}^{\left(i\right)}\left(\text{r}\right)=$$\sum_{k=1}^{N_{e}}\;L_{k}\left(\text{r}\right)\left(e_{3\left(k-1\right)\;+\;1}^{\left(i\right)}\hat{\text{x}}+e_{3\left(k-1\right)\;+\;2}^{\left(i\right)}\hat{\text{y}}+e_{3\left(k-1\right)\;+\;3}^{\left(i\right)}\hat{\text{z}}\right)$, where$N_{e}$is the number of tetrahedrons in the brain,$L_{k}\left(\text{r}\right)=1$in the$k^{\text{th}}$tetrahedron and zero outside it andeis a vector of expansion coefficients and$e_{3\left(k-1\right)\;+\;1}$,$e_{3\left(k-1\right)\;+\;2}$, and$e_{3\left(k-1\right)\;+\;3}$are the average${\text{E}}_{\text{x}}$,${\text{E}}_{\text{y}}$, and${\text{E}}_{\text{z}}$in the$k^{\text{th}}$tetrahedron.

To find the spatial variation of$M^{\left(i\right)}\left(r\right)$, wherei=1,2,...,$N_{m}$, we must orthogonalize the set$\{E_{\text{W}}^{\left(1\right)}\left(\text{r}\right),\;\;{\text{E}}_{\text{W}}^{\left(2\right)}\left(\text{r}\right),...,$${\text{E}}_{\text{W}}^{\left(N_{m}\right)}\left(\text{r}\right)\}$with respect to their inner-product

$$\begin{array}{l} \langle {\text{E}}_{\text{W}}^{\left(i\right)},\;\;{\text{E}}_{\text{W}}^{\left(j\right)}\rangle =\int \Omega \;{\text{E}}_{\text{W}}^{\left(i\right)}\left(\text{r}\right)\;\;\cdot \;\;{\text{E}}_{\text{W}}^{\left(j\right)}\text{dr}=\sum_{k=1}^{N_{e}}V_{k}\sum_{\alpha =1}^{3}e_{3\left(k-1\right)\;+\;\alpha }^{\left(i\right)}e_{3\left(k-1\right)\;+\;\alpha }^{\left(j\right)};\;\\ \;\;\;\;i,j=1,2,...,N_{m}. \end{array}$$ (5)

The above establishes an equivalence between the inner-product of two E-fields and the dot-product of two column vectors of a matrix$\underset{3N_{e}\times \;\;N_{m}}{\text{Z}}$with entries${\text{Z}}_{3\left(k-1\right)\;+\;\alpha ,i}=\sqrt{V_{k}}e_{3\left(k-1\right)\;+\;\alpha }^{\left(i\right)}$, where$k=1,2,...,N_{e}$,α=1,2,3,$i=1,2,...,N_{m}$, and$V_{k}$is the volume of the$k^{\text{th}}$tetrahedron. To find orthonormal mode functions fromZwe first compute its singular value decomposition (SVD)$\text{Z}=\text{US}{\text{V}}^{\text{T}}$. The modes are defined as

$$\begin{array}{l} {\text{M}}^{\left(i\right)}\left(\text{r}\right)=\sum_{k=1}^{N_{e}}\frac{L_{k}\left(\text{r}\right)}{\sqrt{V_{k}}}\left({\text{U}}_{3\left(k-1\right)\;+\;1,i}\hat{\text{x}}+{\text{U}}_{3\left(k-1\right)\;+\;2,i}\hat{\text{y}}+{\text{U}}_{3\left(k-1\right)\;+\;3,i}\hat{\text{z}}\right),\\ \;\;\;\;\;\;\;\;\;\;\;\;\;i=1,2,...,N_{m}, \end{array}$$ (6)

where${\text{U}}_{3\left(k-1\right)\;+\;\alpha ,i}$is the$i^{\text{th}}$column and${\left(3\left(k-1\right)+\alpha \right)}^{\text{th}}$row entry ofU.

### 2.3 Evaluation of coefficients ${\text{a}}^{\left(\text{i}\right)}$

In this section, we provide details of the numerical implementation used to compute the coefficients$a^{\left(i\right)}$.

The expansion coefficients can be computed from${\text{E}}_{\text{TMS}}\left(\text{r}\right)$in the brain and${\text{M}}^{\left(i\right)}\left(\text{r}\right)$by evaluating the following inner product integral

$$a^{\left(i\right)}=\langle {\text{M}}^{\left(i\right)},{\text{E}}_{\text{TMS}}\rangle =\int \Omega {\text{M}}^{\left(i\right)}\left(\text{r}\right)\;\;\cdot \;\;{\text{E}}_{\text{TMS}}\left(\text{r}\right)\text{dr}.$$ (7)

**Fig. 2.**
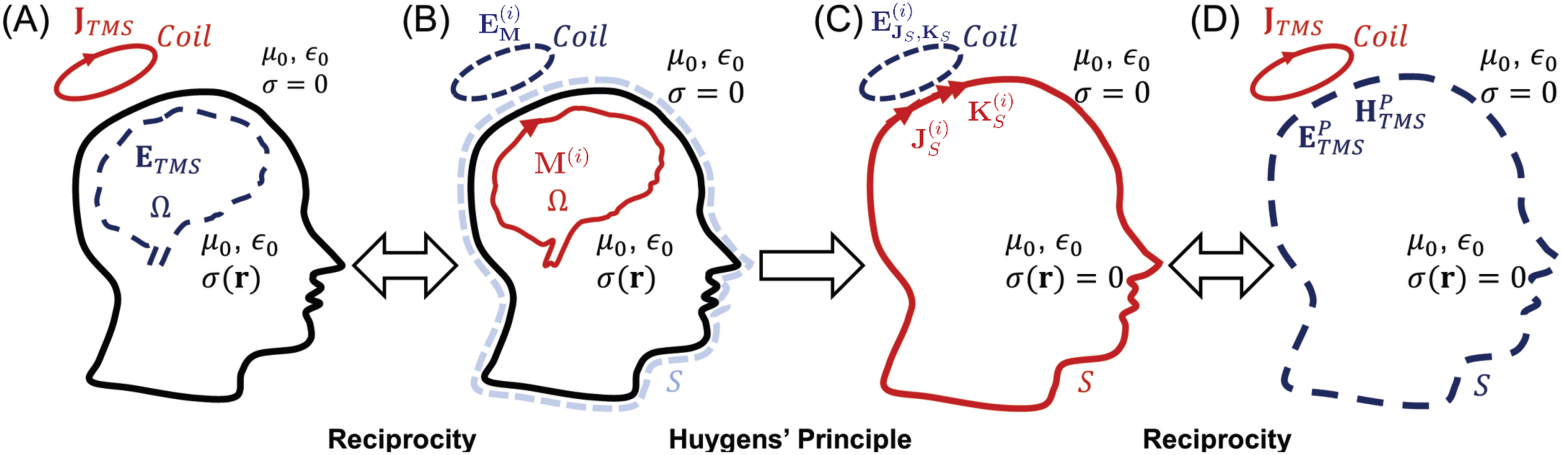
(A) The expansion coefficients can be computed from the induced E-field$\left({\text{E}}_{\text{TMS}}\left(\text{r};t\right)\right)$in the brain due to the coil electric current density outside the scalp$\left({\text{J}}_{\text{TMS}}\left(\text{r};t\right)\right)$. (B) Electromagnetic Reciprocity dictates that the expansion coefficients can also be computed by determining the E-field induced on the coil by mode sources in the brain. (C) According to Huygens’s principle, the fields outside the head generated by the mode sources in the brain can be represented as arising from equivalent electric and magnetic currents on Huygens’s surface. (D) Reciprocity dictates that the expansion coefficients can be computed from the primary E-fields and H-fields on Huygens’s surface induced by the coil.

However, this requires apriori knowledge of TMS-induced E-field in the head. An alternate form ofEquation. (7)can be found by invoking the reciprocity principle. First, we define a new scenario with a mode ($M^{\left(i\right)}\left(\text{r}\right)$) as an electric current source (Fig. 2B). As shown in section 6.3 of theSupplementary FileandGomez et al. (2021), the reciprocity principle dictates that

$$\begin{array}{l} \int_{\Omega }\text{I}\left(t\right){\text{M}}^{\left(i\right)}\left(\text{r}\right)\cdot {\text{I}}^{\prime }\left(t\right){\text{E}}_{\text{TMS}}\left(\text{r}\right)\text{d}\text{r}\\ \;\;\;=\int Coil{\text{I}}^{\prime }\left(t\right){\text{E}}_{\text{M}}^{\left(i\right)}\left(\text{r}\right)\cdot \text{I}\left(t\right){\text{J}}_{\text{TMS}}\left(r\right)\text{d}\text{r}=\text{I}\left(t\right){\text{I}}^{\prime }\left(t\right)a^{\left(i\right)}, \end{array}$$ (8)

where,${\text{J}}_{\text{TMS}}\left(\text{r};t\right)=\text{I}\left(t\right){\text{J}}_{\text{TMS}}\left(\text{r}\right)$is the electric current density in the coil outside the head and${\text{E}}_{\text{M}}^{\left(i\right)}\left(\text{r};t\right)={\text{I}}^{\prime }\left(t\right){\text{E}}_{\text{M}}^{\left(i\right)}\left(\text{r}\right)$is the E-field generated by the impressed mode current sources$\text{I}\left(t\right){\text{M}}^{\left(i\right)}\left(\text{r}\right)$.Equation (8)implies that the integral of the TMS-induced E-field times the mode function is equal to the integral of the E-fields induced by the modes as currents times the TMS coil electric currents. For a detailed mathematical proof and discussion about the reciprocity principle in quasi-static scenarios, the readers are referred to section 6.3 in theSupplementary FileandPlonsey (1972);Hasan et al. (2023);Gomez et al. (2021)and theSupplementary FileofGomez et al. (2021).

Evaluating$a^{\left(i\right)}$by using the reciprocity principle inEquation (8)still requires the calculation of${\text{E}}_{\text{M}}^{\left(i\right)}\left(\text{r}\right)$due to all mode current sources that are inside the head. To reduce the computation, we utilize Huygens’s principle (Balanis, 2012) to replace the mode current distributions${\text{M}}^{\left(i\right)}\left(\text{r};t\right)$by equivalent electric current distributions${\text{J}}_{\text{S}}^{\left(i\right)}\left(\text{r};t\right)=\text{I}\left(t\right){\text{J}}_{\text{S}}^{\left(i\right)}\left(\text{r}\right)$and magnetic current distributions${\text{K}}_{\text{S}}^{\left(i\right)}\left(\text{r};t\right)={\text{I}}^{\prime }\left(t\right){\text{K}}_{\text{S}}^{\left(i\right)}\left(\text{r}\right)$on the Huygens’s surface (S). Both of these current distributions generate E-field${\text{E}}_{{\text{J}}_{\text{S}},{\text{K}}_{\text{S}}}^{\left(i\right)}\left(\text{r};t\right)$everywhere. Outside of the head,${\text{E}}_{\text{M}}^{\left(i\right)}\left(\text{r};t\right)$is equal to${\text{E}}_{{\text{J}}_{\text{S}},{\text{K}}_{\text{S}}}^{\left(i\right)}\left(\text{r};t\right)$(Fig. 2B,2C). Therefore,Equation (8)becomes

$$\begin{array}{l} \text{I}\left(t\right){\text{I}}^{\prime }\left(t\right)a^{\left(i\right)}=\int Coil{\text{I}}^{\prime }\left(t\right){\text{E}}_{\text{M}}^{\left(i\right)}\left(\text{r}\right)\;\;\cdot \;\;\text{I}\left(t\right){\text{J}}_{\text{TMS}}\left(\text{r}\right)\text{d}\text{r}\\ \;\;\;\;\;\;\;\;\;\;\;\;\;\;\;\;\;=\int_{Coil}{\text{I}}^{\prime }\left(t\right){\text{E}}_{{\text{J}}_{\text{S}},{\text{K}}_{\text{S}}}^{\left(i\right)}\left(\text{r}\right)\;\;\cdot \;\;\text{I}\left(t\right){\text{J}}_{\text{TMS}}\left(\text{r}\right)\text{d}\text{r}. \end{array}$$ (9)

As a next step, we invoke the reciprocity principle again (Fig. 2C,2D), and this results in the following

$$\begin{array}{l} \begin{array}{c} \begin{array}{l} \text{I}\left(t\right){\text{I}}^{\prime }\left(t\right)a^{\left(i\right)}=\int_{Coil}{\text{I}}^{\prime }\left(t\right){\text{E}}_{{\text{J}}_{\text{S}},{\text{K}}_{\text{S}}}^{\left(i\right)}\left(\text{r}\right)\;\;\cdot \;\;\text{I}\left(t\right){\text{J}}_{\text{TMS}}\left(\text{r}\right)\text{d}\text{r}, \end{array} \end{array}\\ =\text{I}\left(t\right){\text{I}}^{\prime }\left(t\right)\left[\int_{S}{\text{E}}_{\text{TMS}}^{\text{P}}\left(\text{r}\right)\;\;\cdot \;\;{\text{J}}_{\text{S}}^{\left(i\right)}\left(\text{r}\right))\text{d}\text{r}-\int_{S}{\text{H}}_{\text{TMS}}^{\text{P}}\left(\text{r}\right)\;\;\cdot \;\;{\text{K}}_{\text{S}}^{\left(i\right)}\left(\text{r}\right)\text{d}\text{r}\right]. \end{array}$$ (10)

The last equality involves the TMS coil primary fields because the equivalent scenario (Fig. 2C) has the equivalent surface (Huygens’s surface) current distributions generating fields in empty space (details in section 6.5 of theSupplementary File). For detailed proof ofEquation (10), the readers are referred to section 6.6 of theSupplementary File. The Huygens’s surface (S) is chosen to be the same as the fictitious surface insection 2.2. A single-point Gaussian quadrature is used to determine$a^{\left(i\right)}$. This results in the following

$$a^{\left(i\right)}\approx \sum_{j=1}^{N_{d}}A_{j}\left[{\text{E}}_{\text{TMS}}^{\text{P}}\left({\text{r}}_{j}\right)\;\;\cdot \;\;{\text{J}}_{\text{S}}^{\left(i\right)}\left({\text{r}}_{j}\right)\;\;-\;\;{\text{H}}_{\text{TMS}}^{\text{P}}\left({\text{r}}_{j}\right)\;\;\cdot \;\;{\text{K}}_{\text{S}}^{\left(i\right)}\left({\text{r}}_{j}\right)\right],$$ (11)

where$A_{j}$and${\text{r}}_{j}$are the area and the center position of the$j^{\text{th}}$triangle. To evaluate$a^{\left(i\right)}$numerically, we need to first determine the equivalent electric and magnetic currents on the Huygens’s surface for each of the impressed currents${\text{M}}^{\left(i\right)}\left(\text{r}\right)$, which are described in the next section.

### 2.4 Evaluation of Huygens’s surface current densities

According to the surface equivalence principle (section 7.8 inBalanis (2012)), the fields generated by${\text{M}}^{\left(i\right)}\left(\text{r};t\right)$outside of the surfaceScan be represented as being generated by$J_{S}^{\;\;\;\;(i)}\left(\text{r};t\right)$and$K_{S}^{\;\;\;\;(i)}\left(\text{r};t\right)$. These current densities will generate electric fields

$${\text{I}}^{\prime }\left(t\right){\text{E}}_{{\text{J}}_{\text{S}},{\text{K}}_{\text{S}}}^{\left(i\right)}\left(\text{r}\right)\begin{array}{c} =\{\begin{array}{cc} 0 & ;\text{r}\in \text{inside}S\\ {\text{I}}^{\prime }\left(t\right){\text{E}}_{\text{M}}^{\left(i\right)}\left(\text{r}\right) & ;\text{r}\in \text{outside}S \end{array} \end{array},$$ (12)

and magnetic fields

$$\begin{array}{cc} \text{I}\left(t\right){\text{H}}_{{\text{J}}_{\text{S}},{\text{K}}_{\text{S}}}^{\left(i\right)}\left(\text{r}\right) & =\{\begin{array}{cc} 0 & ;\text{r}\in \text{inside}S\\ \text{I}\left(t\right){\text{H}}_{\text{M}}^{\left(i\right)}\left(\text{r}\right) & ;\text{r}\in \text{outside}S \end{array} \end{array}.$$ (13)

Note that, the fields inside the surfaceSinEquations (12)and(13)are chosen to be zero inside the head to enable the replacement of the conductive head with free-space Furthermore, the fields have to satisfy the electromagnetic boundary conditions. Plugging in the electromagnetic boundary conditions onSfor the equivalent scenario (Fig. 2C) results in (Equation (7-43a) and (7-43b) inBalanis (2012))

$$\begin{array}{cc} J_{S}^{\;\;\;\;(i)}\left(\text{r};t\right) & =\hat{\text{n}}\times \left(\text{I}\left(t\right){\text{H}}_{\text{M}}^{\left(i\right)}\left(\text{r}\right)-0\right),\\ K_{S}^{\;\;\;\;(i)}\left(\text{r};t\right) & =-\hat{\text{n}}\times \left({\text{I}}^{\prime }\left(t\right){\text{E}}_{\text{M}}^{\left(i\right)}\left(\text{r}\right)-0\right). \end{array}$$ (14)

The spatial variation of the currents$J_{S}^{\;\;\;\;(i)}\left(\text{r}\right)$and$K_{S}^{\;\;\;\;(i)}\left(\text{r}\right)$can be determined from${\text{H}}_{\text{M}}^{\left(i\right)}\left(\text{r}\right)$and${\text{E}}_{M}^{\left(i\right)}\left(\text{r}\right)$, respectively.Balanis (2012)andPeterson et al. (1998)provide more detailed explanations of the surface equivalence principle and Huygens’s principle for interested readers (section 7.8 inBalanis (2012)). A detailed proof ofEquation (14)that followsTai (1998)’s approach is provided in sections 6.4-6.5 of theSupplementary Filefor interested readers.

Each mode current generates an E-field inside and outside the head. The E-field inside the head is due to a scalar potential generated by the mode current. The E-field outside the head has a negligible scalar potential component and is equal to the time-derivative of the magnetic vector potential generated by the conduction currents and mode currents. Therefore, the spatial variation of the E-field on the surfaceSis (Gomez et al., 2020a,^2021^;Hasan et al., 2023;D. Wang et al., 2023)

$$E_{M}^{\left(i\right)}(r)=\frac{\mu_{0}}{4\pi }\int_{Head}\frac{M^{\left(i\right)}(r')+\sigma (r')E_{M}^{\left(i\right)}(r')}{\left||r-r'|\right|}dr''r\in S,$$ (15)

whereHeadis the whole head region. The H-field is just the curl of the magnetic vector potential (i.e. Biot-Savart applied to the conduction and mode currents) and its spatial variation is

$${\text{H}}_{\text{M}}^{\left(i\right)}\left(\text{r}\right)=\frac{1}{4\pi }\int_{Head}\frac{\left[{\text{M}}^{\left(i\right)}\left({\text{r}}^{\prime }\right)+\sigma \left({\text{r}}^{\prime }\right){\text{E}}_{\text{M}}^{\left(i\right)}\left({\text{r}}^{\prime }\right)\right]\times \left(\text{r}-{\text{r}}^{\prime }\right)}{{\left\|\text{r}-{\text{r}}^{\prime }\right\|}^{3}}\text{d}{\text{r}}^{\prime };\text{r}\in S$$ (16)

To evaluateEquations (15and16), we first need to determine${\text{E}}_{\text{M}}^{\left(i\right)}\left(\text{r}\right)$in the head (procedure shown inFig. 3). This is done by applying an FEM procedure to$\nabla \cdot \sigma \left(\text{r}\right){\text{E}}_{\text{M}}^{\left(i\right)}\left(\text{r}\right)=\nabla \cdot {\text{M}}^{\left(i\right)}\left(\text{r}\right)$(available online atGomez et al. (2020a)). The integrals inEquations (15and16) are approximated by applying a single point Gaussian quadrature rule for each tetrahedron in the head mesh and are rapidly evaluated using the Fast Multipole Method library (Greengard & Gimbutas, 2012).

**Fig. 3.**
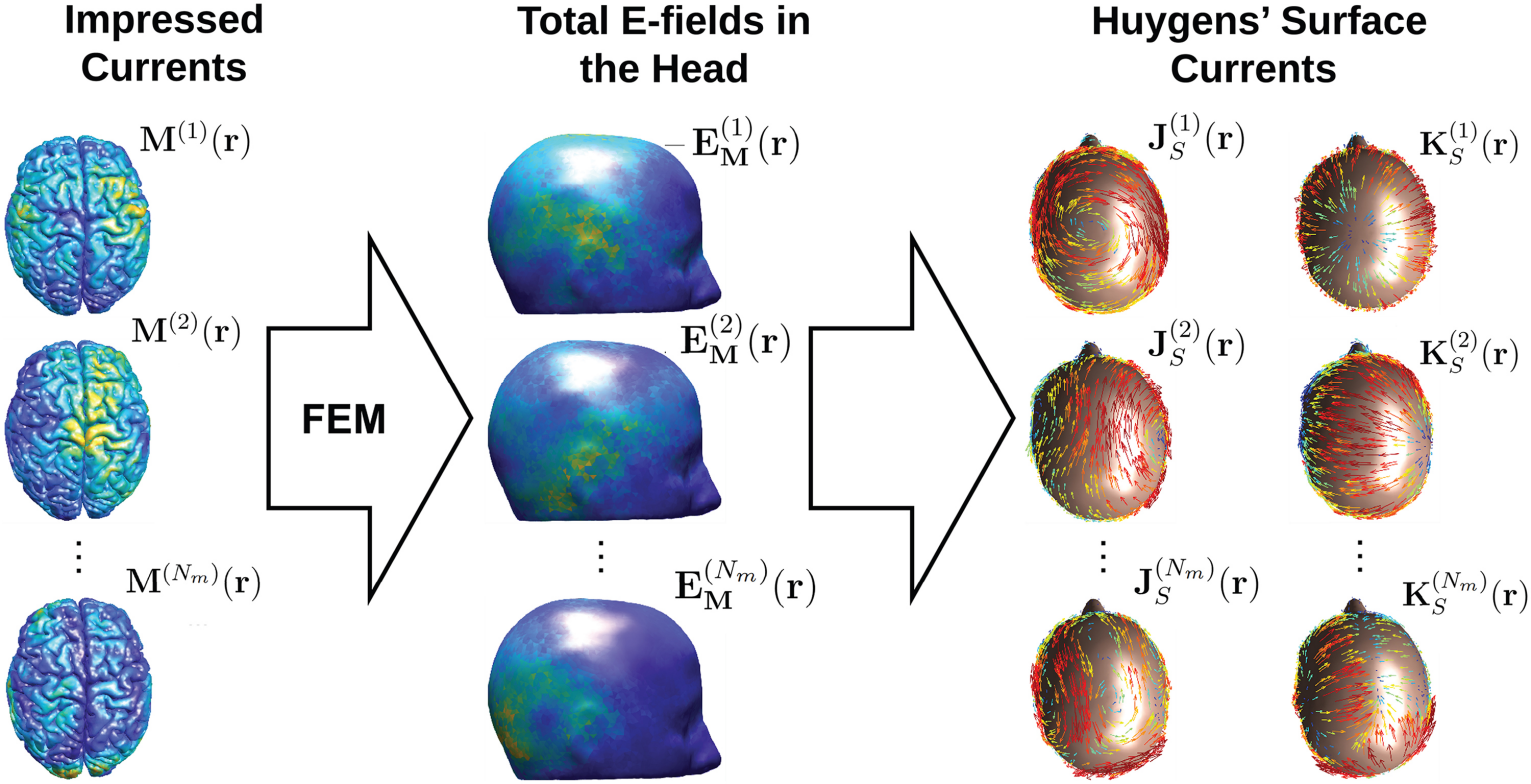
E-fields generated by the individual realization of the orthonormal mode functions or impressed currents$\left({\text{M}}^{\left(i\right)}\left(r\right)\right)$are evaluated on the Huygens’s surface. Then, the electric and magnetic currents are calculated using the reciprocity principle on Huygens’s surface.

### 2.5 Fast evaluation of primary fields due to TMS coils

Evaluation of$a^{\left(i\right)}$and the TMS-induced E-field requires computation of the primary fields generated by the TMS coil on Huygens’s surface. As such, these must be computed in real-time each time the coil moves. Here, we adopt the approach proposed inDaneshzand et al. (2021);Thielscher et al. (2015)that leverages the fact that the primary fields are functions of position relative to the coil (i.e., translational invariance). As a result, the primary fields are rapidly computed by interpolating samples on a 3D Cartesian grid using a process described next.

**Fig. 4.**
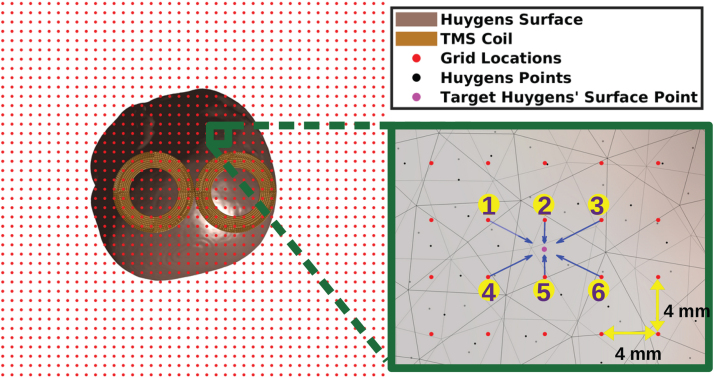
Inverse relative transformation of the Huygens’s surface with respect to the TMS coil inside the interpolation grid points (red). The right figure shows an illustration of the multi-linear interpolation process for an exemplary targeted Huygens’s surface node (pink), where the primary field is interpolated by the nearby grid points (numbered 1–6).

Here, we assume a reference coil placement flat on and centered about the x-y plane. The primary fields are sampled on a 3D grid with 4 mm grid spacing with 822,000 grid points (Fig. 4). This grid spacing empirically was found to result in an interpolation error of the order of$10^{-3}\%$. Results of interpolation errors for various grid spacing are given in theSupplementary File.

The grid is large enough to contain the whole Huygens’s surface for any relative placement with respect to the coil. Coil placements are defined as coordinate transformation (i.e., a rotation and translation) to the coil relative to the reference coil placement as

$$\text{T}=\left[\begin{array}{cc} {\text{R}}_{c} & {\text{T}}_{0}\\ \text{O} & 1 \end{array}\right],$$

where${\text{R}}_{c}$is a3×3rotation matrix and${\text{T}}_{0}$is a3×1translation vector. Applying the transformationTto the coil is equivalent to applying${\text{T}}^{-1}$to the Huygens’s surface. We apply the transformation${\text{T}}^{-1}$to the subjects’ Huygens’s surface instead of the transformationTto the coil. This avoids the need to translate the grid that has822,000grid points and only requires translating120,000Huygens’s surface nodes. Furthermore, this enables us to use the computationally efficient standard multi-linear interpolation method (Kreyszig, 1972). In the standard multi-linear interpolation scheme, the E-field is interpolated at the center of Huygens’s surface triangular facets using the nearby grid point values (where the E-fields have already been pre-computed), and the interpolation is performed along$\hat{x},\hat{y},$and$\hat{z}$, separately, using the ‘interpn()’ functionality in MATLAB (MATLAB, 2022).

### 2.6 Summary of the real-time TMS pipeline

This section summarizes the offine mode and surface equivalent current calculation stage and the real-time E-field calculation stage. Algorithm 1 summarizes the critical steps for computing the mode functions. Algorithm 2 describes the four fundamental steps to calculate the TMS-induced E-field in theROIin real-time while the modes and primary fields are already pre-computed. The algorithms were implemented in MATLAB 2022a (MATLAB, 2022) with built-in GPU functionalities from the ‘Parallel Computing Toolbox’. The current implementation of the real-time stage is on NVIDIA GPUs or any GPU that MATLAB supports. However, the real-time stage requires only two dense matrix-vector multiplications and a multilinear interpolation. As such, it is easily portable to any GPU package with those capabilities.

**Algorithm 1.** Pre-processing stage (Mode and equivalent surface current calculation)

Inputs: number of modes ( $N_{m}$ ), tetrahedron mesh of head model with $N_{e}$ brain ROI tetrahedrons, Huygens’s surface triangle mesh consisting of $N_{d}$ triangles. 1. **White noise current and field generation** for $i=1,2,...,N_{m}$ , (a) Generate ${\text{W}}^{\left(i\right)}({\text{r}}_{j})$ - samples of white noise magnetic current density (randomly weighted magnetic dipoles) at centers of j Huygens’s surface triangles ( $j=1,2,...,N_{d}$ ), where ${\text{r}}_{j}\in S$ . (b) Compute primary E-field, $)\;{\text{E}}_{\text{W}}^{\text{P}\left(i\right)}\left(\text{r}\right)$ , in the head using white noise source samples ${\text{W}}^{\left(i\right)}({\text{r}}_{j})$ via Equation (3) . (c) Solve for the scalar potential $\phi^{\left(i\right)}$ using FEM to solve Equation (4) and compute total E-field in the brain, ${\text{E}}_{\text{W}}^{\left(i\right)}\left(\text{r}\right)=\left\{{\text{E}}_{\text{W}}^{\text{P}\left(i\right)}\left(\text{r}\right)-\nabla \phi^{\left(i\right)}\left(\text{r}\right)\right\}$ . 2. **Orthonormal mode function generation** (a) Construct the matrix $\underset{3N_{e}\times \;N_{m}}{\text{Z}}$ with entries ${\text{Z}}_{3\left(k-1\right)\;+\;\alpha ,i}=\sqrt{V_{k}}e_{3\left(k-1\right)\;+\;\alpha }^{\left(i\right)}$ , where $k=1,2,...,N_{e}$ , α=1,2,3 , and $V_{k}$ is the volume of the $k^{\text{th}}$ tetrahedron. (b) Compute the economic QR decomposition, Z=QR . (c) Compute the SVD of $\text{R}=\tilde{\text{U}}\tilde{\Sigma }{\tilde{\text{V}}}^{\text{T}}$ . (d) Compute the unitary matrix, $\text{U}=\text{Q}\tilde{\text{U}}$ . (e) Compute the mode function ${\text{M}}^{\left(i\right)}\left(\text{r}\right)$ from the matrix U via Equation (6) . 3. **Huygens’s surface current generation** for $i=1,2,...,N_{m}$ , (a) Use FEM to compute E-field, ${\text{E}}_{\text{M}}^{\left(i\right)}\left(\text{r}\right)$ , in the head generated by impressed current ${\text{M}}^{\left(i\right)}\left(\text{r}\right)$ . (b) Compute Huygens’s surface electric current density distribution ${\text{J}}_{\text{S}}^{\left(i\right)}({\text{r}}_{j})=\hat{\text{n}}\times {\text{H}}_{\text{M}}^{\left(i\right)}({\text{r}}_{j})$ and magnetic current density distribution ${\text{K}}_{\text{S}}^{\left(i\right)}({\text{r}}_{j})=-\hat{\text{n}}\times {\text{E}}_{\text{M}}^{\left(i\right)}({\text{r}}_{j})$ at Huygens’s surface triangle centers ( $j=1,2,...,N_{d}$ ) via Equations (14 , 15 , and 16 ).

Note that the induced E-fields$\left({\text{E}}_{\text{W}}^{\left(i\right)}\left(\text{r}\right)\right)$in the brain due to random magnetic sources$\left({\text{W}}^{\left(i\right)}\left(\text{r}\right)\right)$span the TMS-induced E-field space. To find an orthonormal basis set of${\text{E}}_{\text{W}}^{\left(i\right)}\left(\text{r}\right)$, we must perform an SVD onZ. Instead of performing SVD on the matrixZitself, we implement the SVD by doing an economic QR decomposition ofZfollowed by an SVD onRbecause it is more efficient than doing an SVD on the original matrix. Both methods provide the same result, but the latter is more computationally efficient as we perform SVD on a much smaller matrixRthanZ.

**Algorithm 2.** Real-Time E-field Calculation

Inputs: $N_{m}$ orthonormal mode functions ( ${\text{M}}^{\left(i\right)}\left(\text{r}\right);i\in \left\{1,2,...,N_{m}\right\}$ ), $N_{m}$ Huygens’s surface electric and magnetic current distribution ( ${\text{J}}_{\text{S}}^{\left(i\right)}\left(\text{r}\right)$ , ${\text{K}}_{\text{S}}^{\left(i\right)}\left(\text{r}\right)$ ; $i\in \left\{1,2,...,N_{m}\right\}$ ), pre-computed primary electric and magnetic currents [ ${\text{E}}_{\text{TMS}}^{\text{P}}\left(\text{r}\right)$ , $){\text{H}}_{\text{TMS}}^{\text{P}}\left(\text{r}\right)$ ] in the 3D interpolation grid, transformation matrix ( T ) for the coil placement (provided by neuronavigation system). 1. **Huygens’s surface transformation and primary field interpolation** for $j=1,2,...,N_{d}$ , (a) Transform the centers of triangular facets in Huygens’s surface mesh, ${{\text{r}}^{\prime }}_{j}={\text{T}}^{-1}{\text{r}}_{j}$ . (b) Interpolate the primary fields, ${\text{E}}_{\text{TMS}}^{\text{P}}$ and ${\text{H}}_{\text{TMS}}^{\text{P}}$ , at ${{\text{r}}^{\prime }}_{j}$ . 2. **Mode coefficient calculation** for $i=1,2,...,N_{m}$ , Compute $a^{\left(i\right)}\approx \sum_{j=1}^{N_{d}}A_{j}\left[{\text{E}}_{\text{TMS}}^{\text{P}}({\text{r}}_{j})\cdot {\text{J}}_{\text{S}}^{\left(i\right)}({\text{r}}_{j})-{\text{H}}_{\text{TMS}}^{\text{P}}({\text{r}}_{j})\cdot {\text{K}}_{\text{S}}^{\left(i\right)}({\text{r}}_{j})\right]$ 3. Compute the TMS E-field at desired locations using ${\text{E}}_{\text{TMS}}^{\left(N_{m}\right)}\left(\text{r}\right)=\sum_{i=1}^{N_{m}}a^{\left(i\right)}{\text{M}}^{\left(i\right)}\left(\text{r}\right)$ .

### 2.7 Coil and head models

The algorithm is tested on 14 MRI-derived heads and three distinct TMS coil models. The MRI-derived head models are generated from eight distinct subject MRIs. One is the ‘Ernie’ subject included in SimNIBS-3.2 (Thielscher et al., 2015) and the seven others were collected fromLee et al. (2016). The 16 head models are generated using the ‘mri2mesh’ and ‘headreco’ tools in SimNIBS (Nielsen et al., 2018;Windhoff et al., 2013). The ‘mri2mesh’ models comprise 668,000–742,000 nodes and 3.73–4.16 million tetrahedrons. On the other hand, the ‘headreco’ models consist of 528,000–886,000 nodes and 2.87–4.92 million tetrahedrons. The average edge lengths on the cortex, scalp, and Huygens’s surface are 1.4 mm, 1.65 mm, and 1.66 mm, respectively.

We consider only the five homogeneous concentric compartments such as (from inner to outer) white matter, grey matter, cerebrospinal fluid (CSF), skull, and scalp with corresponding conductivity of 0.126, 0.275, 1.654, 0.01, and 0.465 S/m, respectively (Wagner et al., 2004) in all head models. The time required for the generation of each head model was between 20–24 hours using the ‘mri2mesh’ tool and 1.5–2 hours using the ‘headreco’ tool.

During the preprocessing stage (offline stage or, mode calculation stage), we compute the E-fields in the brain (grey matter and white matter) consisting of 1.31–1.84 million tetrahedrons for ‘headreco’ models and 1.55–1.65 million tetrahedrons for ‘mri2mesh’ models. During the real-time stage, we compute the E-field at the barycenter of each triangular facet on the middle grey matter surface (a surface approximately midway into the grey matter (Stenroos & Koponen, 2019)) consisting of 122,000–289,000 triangular elements for ‘headreco’ models and 241,000–284,000 triangular elements for ‘mri2mesh’ models.

Our method is suitable for modeling any TMS coil; to illustrate, we included different coil types and sizes in this study. The Figure-8 coil consists of two9turn concentric circular loops with inner and outer loop diameters of 53 mm and 88 mm, respectively, which matches the 70-mm Figure-8 #31 inDeng et al. (2013)and is approximated with the coil model described inGomez et al. (2020b). The circular and double cone coil models were obtained fromMakarov (2020)and are models of the MagVenture Cool-40 Rat coil and D-B80 coil, respectively. The coils are modeled with electric dipoles. The coil is centered 5 mm directly above and oriented tangent to a scalp landmark. The number of dipoles in Figure-8, MagVenture Cool-40, and D-B80 coils are 193,536, 57,024, and 22,400, respectively. The Huygens’s surface is just 1 mm outward from the scalp. During the grid interpolation, the primary fields are pre-computed on the grid and the grid is right below the coil but large enough to hold the head and the Huygens’s surface.

### 2.8 Error metrics

We performed a benchmark comparison between our real-time algorithm with the conventional$1^{\text{st}}$-order FEM solver. We consider global vector error (GVE) and global magnitude error (GME) as means of comparison between the FEM-calculated E-field$\left({\text{E}}_{\text{TMS}}\left(\text{r}\right)\right)$and the real-time computed E-field$\left({\text{E}}_{\text{TMS}}^{\left(N_{m}\right)}\left(\text{r}\right)\right)$in theROI, defined as follows:

$$\text{GVE}=\frac{∥{\text{E}}_{\text{TMS}}^{\left(N_{m}\right)}\left(\text{r}\right)-{\text{E}}_{\text{TMS}}\left(\text{r}\right)∥}{∥{\text{E}}_{\text{TMS}}\left(\text{r}\right)∥}\times 100\%,$$ (17a)

$$\text{GME}=\frac{\left\|\;\left|{\text{E}}_{\text{TMS}}^{\left(N_{m}\right)}\left(\text{r}\right)\left|-\right|{\text{E}}_{\text{TMS}}\left(\text{r}\right)\right|\;\right\|}{∥{\text{E}}_{\text{TMS}}\left(\text{r}\right)∥}\times 100\%,$$ (17b)

where‖ · ‖denotes the$L^{2}$norm and|·|is the magnitude for the E-field. The real-time solver aims to recreate the FEM predicted field, as a result, the reference E-field (${\text{E}}_{\text{TMS}}\left(\text{r}\right)$) is computed by the in-house built$1^{\text{st}}$-order FEM solver (Gomez et al., 2020a). Unlike the inverse relative transformation and multilinear interpolation in the real-time solver, the primary E-field of the reference solver was computed by direct transformation of the coil with respect to the head. Additionally, for a visual comparison of the E-fields, we consider the point-wise relative errors: local vector error (LVE) and local magnitude error (LME), normalized by the largest E-field magnitude in theROIfrom the FEM solver defined as follows:

$$\text{LVE}=\frac{\left|{\text{E}}_{\text{TMS}}^{\left(N_{m}\right)}\left(\text{r}\right)-{\text{E}}_{\text{TMS}}\left(\text{r}\right)\;\right|}{{\text{max}}_{\text{r}\in ROI}\left|{\text{E}}_{\text{TMS}}\left(\text{r}\right)\right|}\times 100\%,$$ (18a)

$$\text{LME}=\frac{\left|\left|{\text{E}}_{\text{TMS}}^{\left(N_{m}\right)}\left(\text{r}\right)|-\;|{\text{E}}_{\text{TMS}}\left(\text{r}\right)\;\right|\right|}{{\text{max}}_{\text{r}\in ROI}\left|{\text{E}}_{\text{TMS}}\left(\text{r}\right)\right|}\times 100\%.$$ (18b)

For all of our analyses, we perform simulations for modes 100 to 500, with a step size of 50.

## 3 Results

### 3.1 Accuracy of real-time predicted E-fields as a function of modes

In this section, we compare observed errors for all 16 head models and the 70-mm Figure-8 coil model. We randomly select 1000 coil placements for each head model and calculate the errors by comparing them with the reference$1^{\text{st}}$-order FEM solution. The convergence of GME and GVE is shown inFigure 5as a function of the number of modes. For ‘mri2mesh’ models, the mean GME and mean GVE are below 2% at modes (equals matrix rank) of325and450, respectively. For ‘headreco’ models, the required modes are350and475for mean GME and GVE, respectively. There are some outlier errors, primarily corresponding to specific coil placements, which nonetheless remain below 3% for GVE and under 2% for GME at rank500. Here, we used the default ‘headreco’ and ‘mri2mesh’ mesh models whose FEM solution is known to have a GVE near 5% (Nielsen et al., 2018). With 400 modes we observed a maximum GVE and GME error of 4% and 3%, respectively, across all simulations. To ensure that the real-time results are just as accurate as the$1^{\text{st}}$-order FEM, we estimated the error of the$1^{\text{st}}$-order FEM and real-time solutions by using a$2^{\text{nd}}$-order FEM as reference (results are given in theSupplementary File). For 400 modes, the resulting difference in GVE and GME between real-time and$1^{\text{st}}$-order FEM was, on average, 0.17% and 0.14%, respectively (Fig. S4). Furthermore, the GVE and GME across the 16,000 simulations showed that the real-time solution with 400 modes is up to 1.3% (1.7%) and 0.7% (1.1%) more (less) in agreement, respectively, than the$1^{\text{st}}$-order FEM to the$2^{\text{nd}}$-order FEM solution (Fig. S5 and S6), indicating that the real-time results are as accurate as the$1^{\text{st}}$-order FEM ones.

**Fig. 5.**
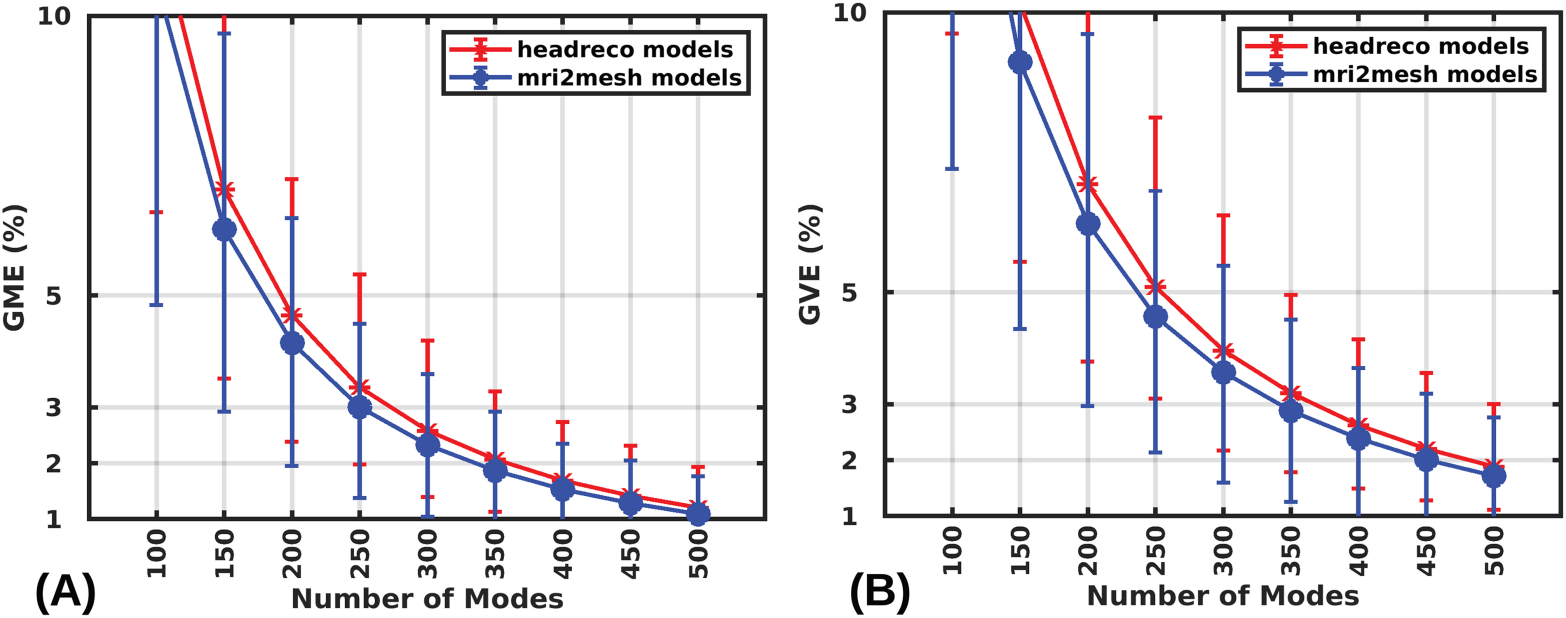
Convergence of GME (A) and GVE (B) as a function of the number of modes for both ‘mri2mesh’ and ‘headreco’ models with a 70-mm Figure-8 coil model. The error distribution for any mode is calculated across 8000 random coil placements (1000 random coil placements over the scalp of each of the eight head models).

### 3.2 Effect of coil model on error convergence

Here, consider the relative accuracy performance of the method for three distinct coil models 70-mm Figure-8, MagVenture D-B80 coil, and Cool-40 Rat coil. We consider the sample mean error over 1000 random coil placements over the scalp on each of the 16 head models and use the$1^{\text{st}}$-order FEM solution as a reference. The mean GME and the mean GVE are shown inFigure 6. The mean GME is below 2% for ‘mri2mesh’ (as well as ‘headreco’) head models at ranks above325,425, and375(350,375, and375) for the Figure-8, D-B80, and Cool-40 coils, respectively. Due to the unique bending shape, the D-B80 coil has an E-field that has more fine features relative to the others, thereby, requiring comparatively more modes for its expansion. The mean GVE is below 2% for ‘mri2mesh’ (‘headreco’) head models at ranks above450,550, and475(475,525, and500) for the Figure-8, D-B80, and Cool-40 coils, respectively. All coils exhibit similar errors. However, compared to the D-B80 and Cool-40 coils, the Figure-8 coil model converges to the 2% error limit with fewer modes.

**Fig. 6.**
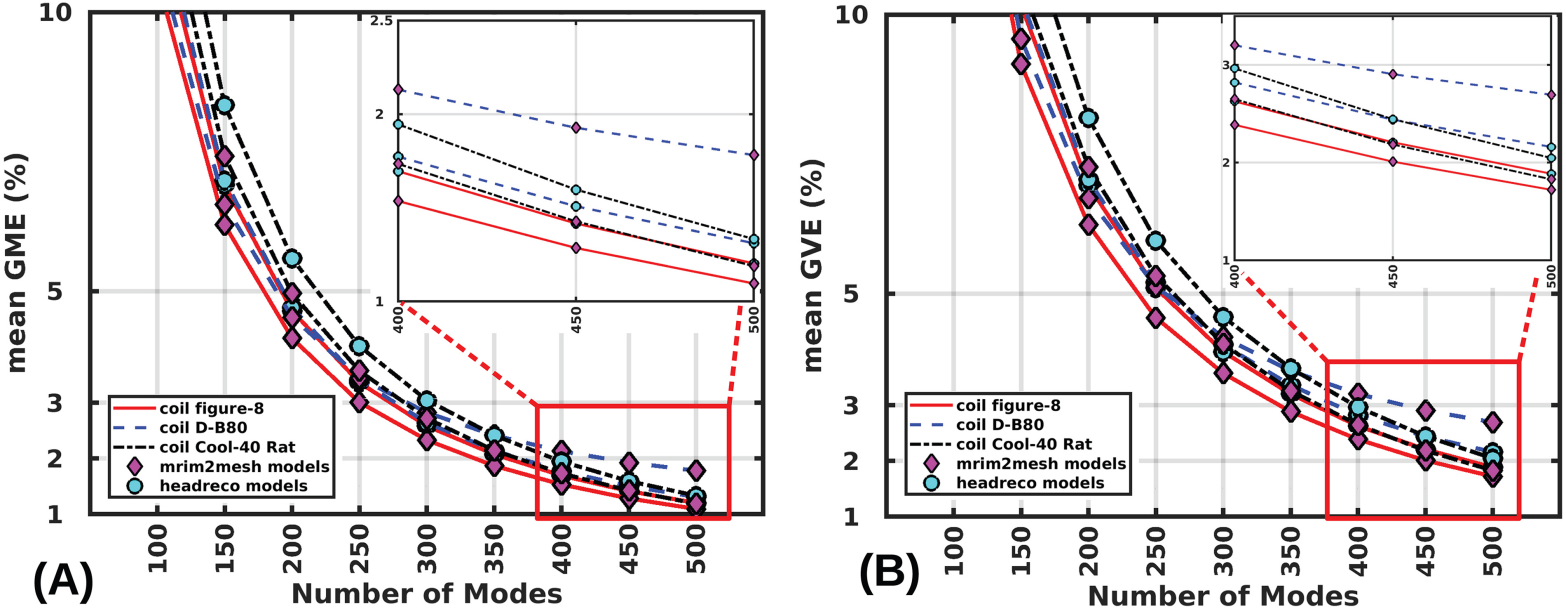
Convergence of mean GME (A) and mean GVE (B) as a function of the number of modes for both ‘mri2mesh’ and ‘headreco’ models with three coils (70-mm Figure-8, MagVenture D-B80 coil, and Cool-40 Rat coil). The mean error for any mode is calculated across 16,000 random coil placements (1000 random coil placements over the scalp of each of the 16 head models from 8 subjects). The inset of each plot shows the errors for the higher number of modes (400–500).

**Fig. 7.**
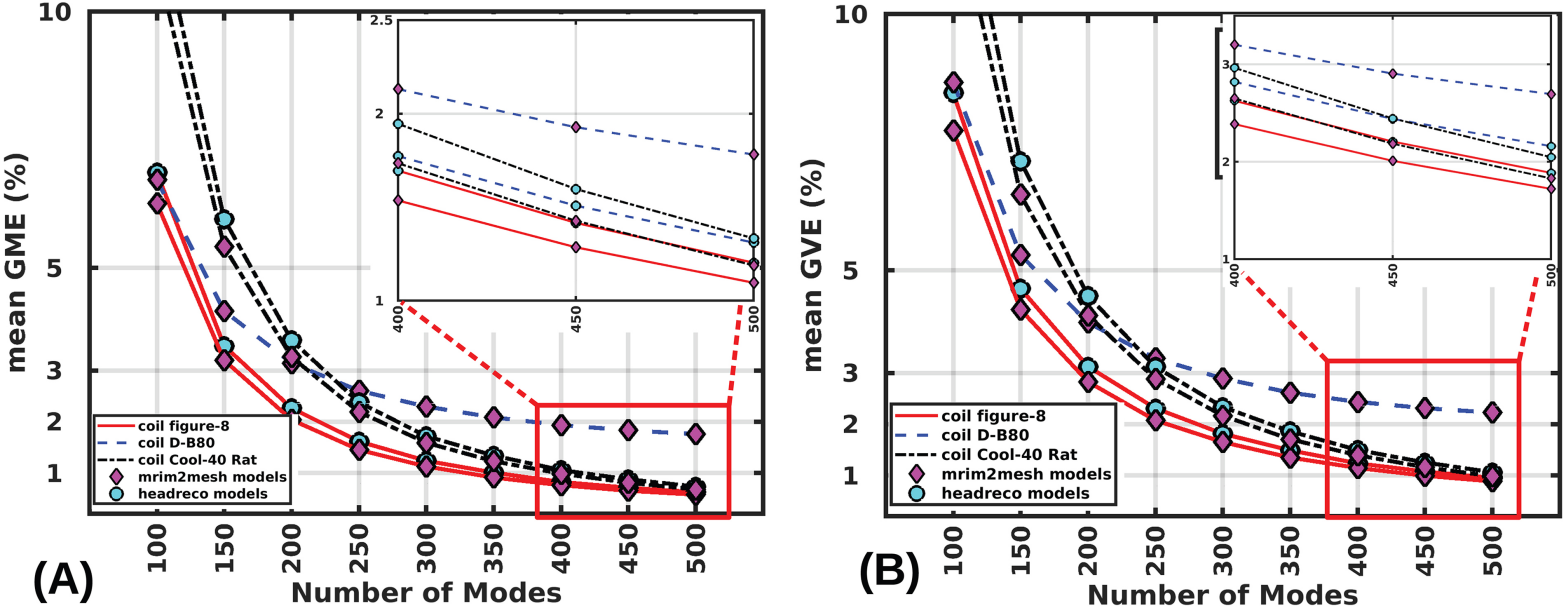
Convergence of mean GME (A) and mean GVE (B) for E-fields in theROIabove 70% of the maximum E-field as a function of the number of modes for both ‘mri2mesh’ and ‘headreco’ models with three coils (70-mm Figure-8, MagVenture D-B80 coil, and Cool-40 Rat coil). The mean error for any mode is calculated across 16,000 random coil placements.

Figure 7shows the convergence of error metrics as a function of modes for theROIregions where${\text{E}}_{\text{TMS}}\left(\text{r}\right)\geq 0.7{\text{max}}_{\text{r}\in ROI}\left({\text{E}}_{\text{TMS}}\left(\text{r}\right)\right)$.)). In this scenario, it requires 350 (500) modes for mean GME (GVE) to converge to the 2% error limit (considering all coil models and all head model types). This is in contrast to 425 (550) (Fig. 6) for E-fields across the whole cortex.

### 3.3 E-field visualization

**Fig. 8.**
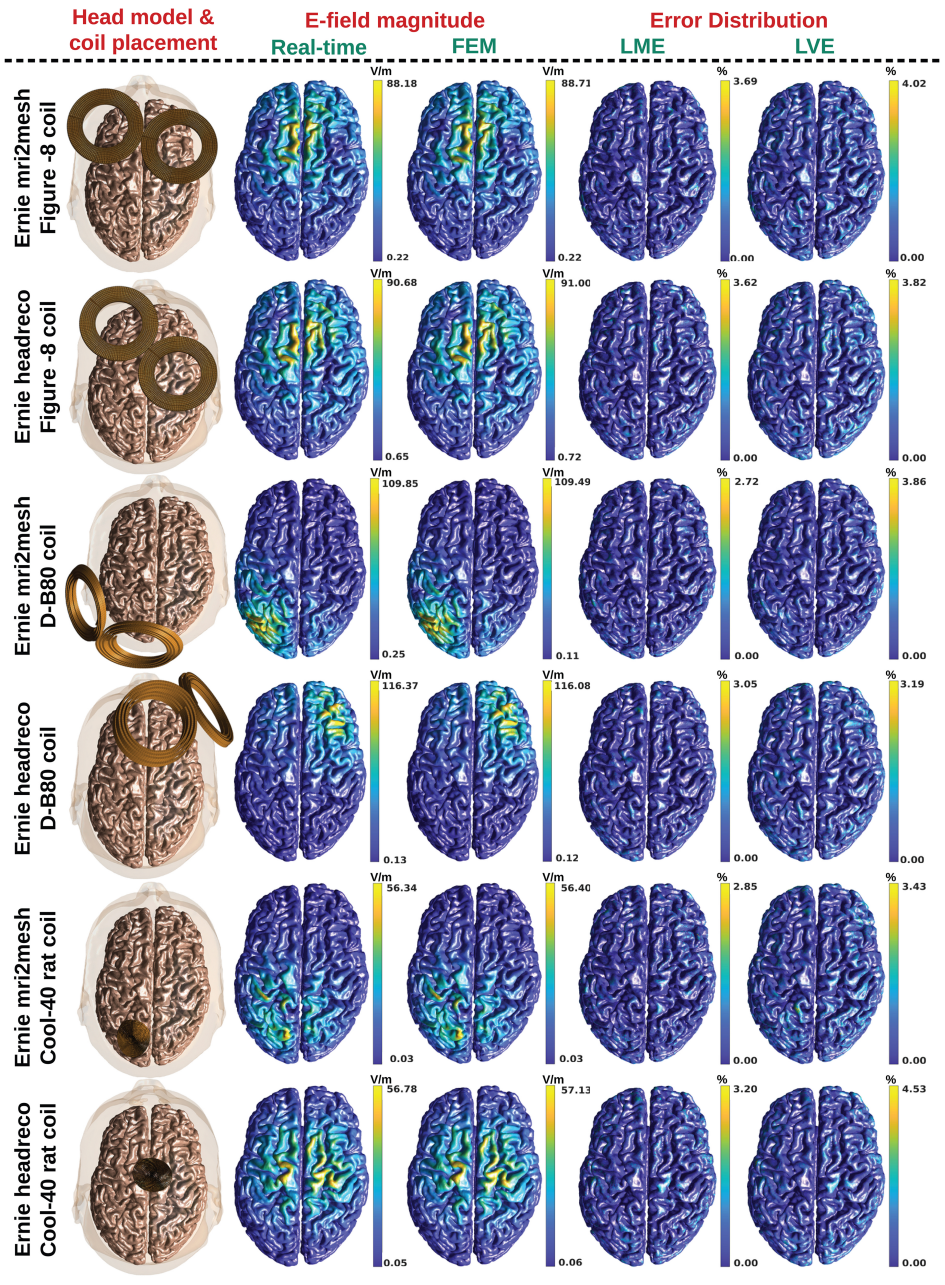
Illustration of the real-time TMS-induced peak E-field ($2^{\text{nd}}$column) and FEM-induced E-field ($3^{\text{rd}}$column) on the middle grey matter surface for randomly chosen coil placements ($1^{\text{st}}$column) over the scalp of SimNIBS 3.2’s ‘Ernie’ head model. The last two columns show the local error distributions (LME and LVE) over the middle grey matter surface. Note: All results assume a coil current peak time-derivative of$6.6\times 10^{7}$A/s to achieve TMS-induced level E-fields

In this section, several exemplary simulation results of coil placements over the scalp of the ‘Ernie’ head model are shown.Figure 8shows the specific coil placement over the scalp, the associated E-field induced on the middle grey matter surface computed in Real-time and FEM solvers, and the corresponding LME and LVE distributions. The E-field distributions predicted in real-time and FEM are visually indistinguishable. Furthermore, the peak E-field is the same up to 0.65 V/m in all cases shown. The maximum LME for each scenario (top to bottom) are 3.7%, 3.6%, 2.7%, 3.1%, 2.9%, and 3.2%, whereas the corresponding maximum LVE is 4%, 3.8%, 3.9%, 3.2%, 3.4%, and 4.5%, respectively. These results indicate that the real-time predicted E-field distributions are equally valid to the FEM$1^{\text{st}}$-order solutions, which have an LME of about 5%.

### 3.4 Computational run-time and memory requirements

**Fig. 9.**
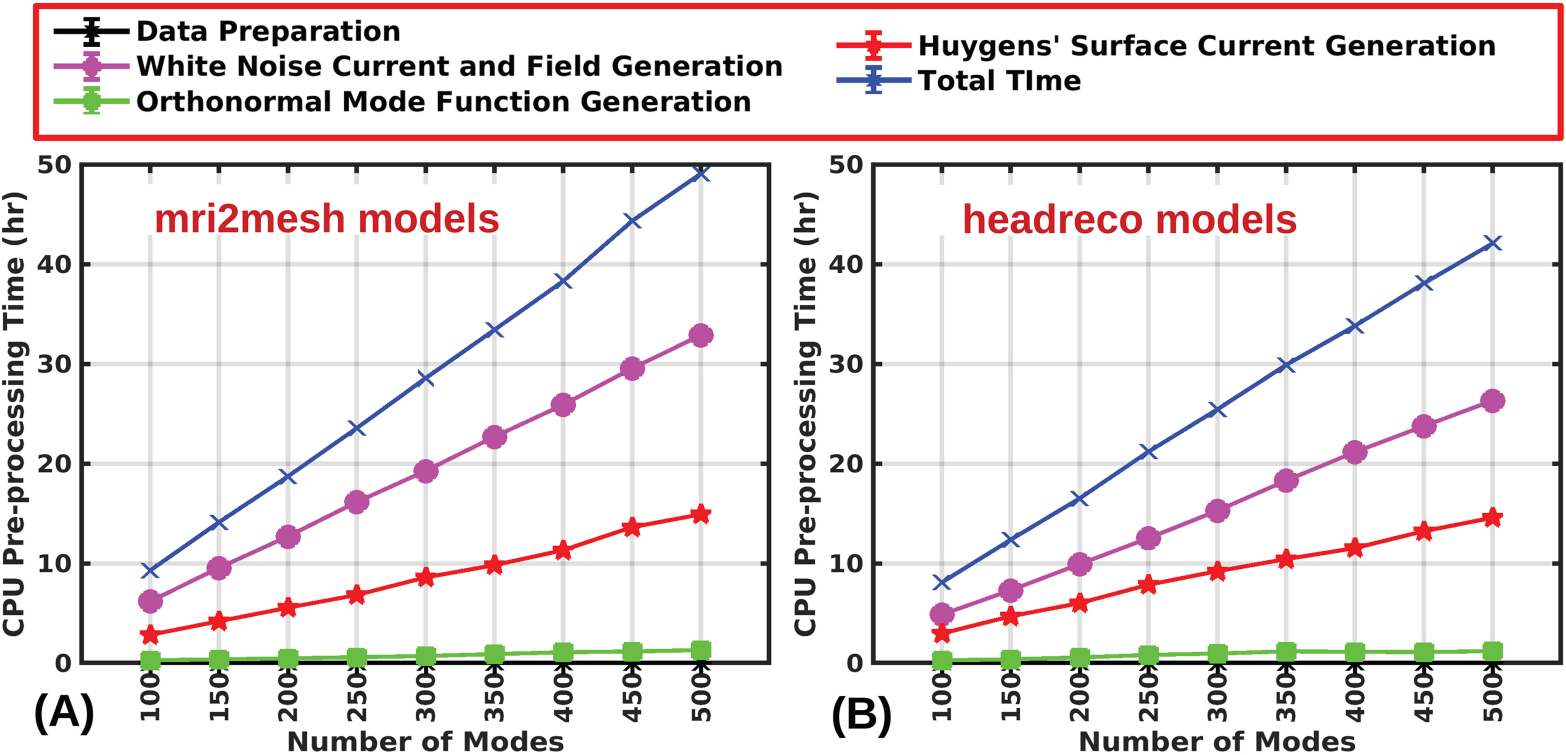
Mean computational time for pre-processing stage (mode and field generation stage) for ‘mri2mesh’ models (A) and ‘headreco’ models (B). At any rank (mode), the time is calculated across eight head models from eight subjects.)

Here we consider single-precision arithmetic during real-time computation, as the results remained unchanged up to seven digits relative to double-precision.Figure 9Aand9Bshow the mean mode calculation time (pre-processing time) as a function of the number of modes across eight ‘mri2mesh’ models and eight ‘headreco’ models, respectively. The pre-processing stage is computed using an AMD Rome 2.0 GHz CPU. On average the pre-processing time required to generate400modes is38hours and34hours for ‘mri2mesh’ and ‘headreco’ models, respectively. The total computational runtime is 2 FEM runs per mode. Our single-threaded implementation requires, on average, 3 minutes of computation time per simulation in an AMD Rome 2.0 GHz processor. The required pre-processing computations could be significantly sped up by using multiple threaded FEM solvers.

**Fig. 10.**
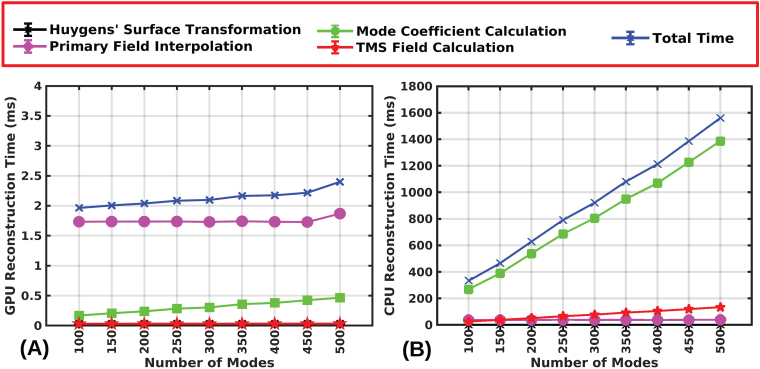
E-field reconstruction time in GPU (A) and CPU (B) as a function of the number of modes. For any mode, the time is calculated across 48,000 random coil placements (1000 random coil placements over the scalp of each head model from each subject for each coil model.)

The core FEM and reciprocity integrals are implemented in C complied for MATLAB and the real-time stage was implemented in MATLAB with GPU functionalities.Figure 10shows the computational reconstruction time as a function of the number of modes for a GPU and CPU, respectively. The above result was obtained by running 48,000 simulations across the 16 head models and 3 coil models. The mean reconstruction time to predict the E-field over the intermediate grey matter-white matter surface for a fixed coil placement is 2.2 ms for 400 modes in an NVIDIA RTX 3080 GPU with a maximum time of 3.8 ms. The first step of the real-time TMS (coordinate transformation of the Huygens’s surface) takes only 0.03 ms, whereas the CPU takes 0.9 ms (a30times speed-up). The second step (multi-linear interpolation of primary fields) takes 1.7 ms in a GPU and 37.40 ms in a CPU. In other words, the GPU requires22times less run-time than the CPU. The mode coefficient calculation is completed in 0.4 ms in a GPU and 1100 ms in a CPU (i.e., a2750times faster). Finally, the fourth step (TMS-induced E-field computation) is rapidly computed in 0.03 ms in a GPU versus 105 ms in the CPU (3500times faster). Overall, the mean total time for estimating the TMS-induced E-field using400modes is 2.2 ms in a GPU and 1200 ms in the CPU (550times faster). This ratio could be improved by accelerating Huygens’s surface coordinate transformation.

**Fig. 11.**
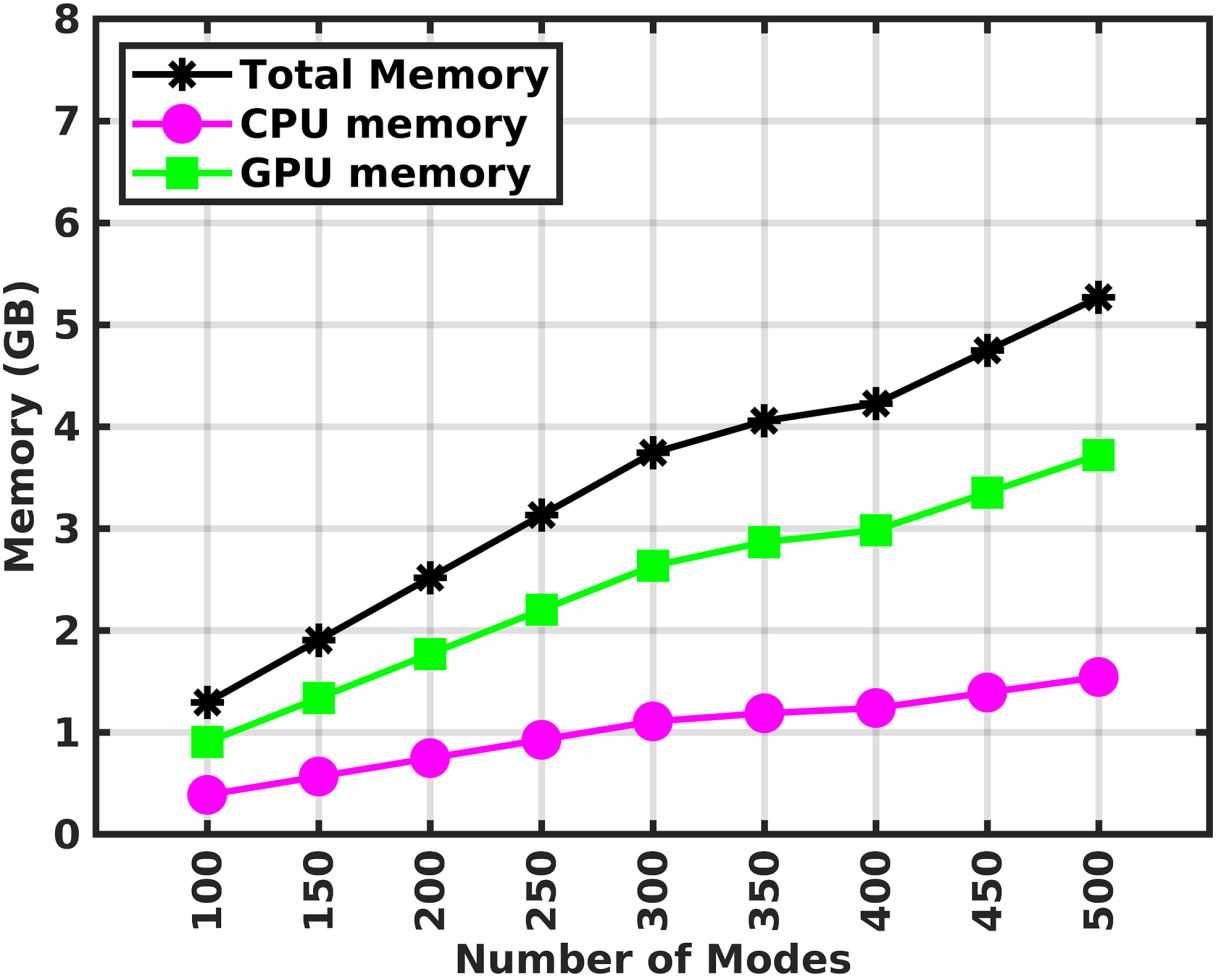
Mean computational memory (in Gigabytes, GB) requirement in a CPU and GPU as a function of the number of modes when the real-time computation is performed in a GPU.

Figure 11shows the required memory in the reconstruction stage for both CPU (AMD Rome CPU, 2.0 GHz) and GPU (NVIDIA RTX 3080-10 GB) across 14 head models. The total required memory in the reconstruction stage is the same for both the GPU and the CPU. The differences in GPU and CPU memory requirements stem from the fact that the Matlab environment requires overhead that is not accounted for in the GPU memory. In other words, the GPU only has all required data structures (e.g., modes, surface currents, and interpolatory primary fields). When the real-time computation is performed in the GPU, the required mean CPU and the GPU memory for 400 modes are 1.3 GigaBytes (GB) and 3 GB, respectively. Additionally, the required mean CPU memory during real-time computation in the same CPU is 4.3 GB. Additionally, section 6.7 in theSupplementary Fileprovides an estimation of the floating point operations (FLOPS) required for the real-time stage.

## 4 Discussion

The real-time TMS-induced E-field approximation method excels in rapidly calculating the E-field over the middle grey matter surface using GPU acceleration, achieving this computation within 4 ms for any coil placement over the scalp of the subject. Our detailed benchmarking analysis indicates that450modes are enough to achieve a mean GME and a mean GVE below 2%. Additionally, with only400modes, the maximum GME and maximum GVE are 2.8% and 4.2%, respectively. Therefore, with400modes, the agreement between the real-time predicted E-fields and$1^{\text{st}}$-order FEM is closer than the expected 5% error of the FEM solution. These results are consistent across multiple subjects, head model construction pipelines, coil types, and coil placements.

In this study, we conducted a benchmark comparison between our real-time algorithm and the$1^{\text{st}}$-order FEM solver, which has a known relative error of about5%(Thielscher et al., 2015). Correspondingly, we observed that the convergence of the mode expansion greatly decelerated below5%. This is likely because, below the FEM solution error threshold, we are increasingly spanning the erroneous part of the solution, which is not expected to be spanned by a small number of modes. We additionally used$2^{\text{nd}}$-order FEM, which results in a more accurate E-field prediction, for a single subject to generate the mode expansion. The results given in theSupplementary Fileindicate that using a more accurate solver (i.e., FEM$2^{\text{nd}}$-order) could result in slightly improved convergence. At the current time, running the$2^{\text{nd}}$-order FEM requires excessive preprocessing time, thereby limiting the applicability of this method. Although not pursued here, this tool could be implemented using efficient implementations of BEM (Makarov et al., 2020) that are more accurate than$1^{\text{st}}$-order FEM (Gomez et al., 2020b) to achieve improved convergence. Furthermore,$1^{\text{st}}$-order FEM is currently the most commonly used tool for TMS E-field dosimetry.

Our method relies on the existence of a small set of modes that span the possible range of E-fields induced by a TMS coil. From spherical solutions, we expect that the total required modes are larger for superficial regions relative to deeper ones. As such, this method could in principle be applied to determine E-fields on more superficial compartments like the CSF, skull, and skin. However, a larger number of modes would likely be necessary in such cases, which could potentially limit its applicability.

The preprocessing stage can be done using any available FEM or BEM code. Using our$1^{\text{st}}$-order in-house FEM solver (Gomez et al., 2020a,2020b), which is optimized for accuracy, requires less than40hours for400modes at 180 s per FEM run. However, a faster implementation of the underlying FEM can speed up the process. For example, the SimNIBS implementation of FEM requires a runtime of 29.8 s, which reduces the total time to 6.6 hours.

The real-time stage requires only under 4 ms (assuming 400 modes) in an NVIDIA RTX 3080 GPU and 1.2 s in an AMD Rome 2.0 GHz CPU to predict the TMS-induced E-field. The reconstruction time is almost independent of the number of modes in a GPU. Therefore, for better accuracy, a higher number of modes can be accommodated in a higher-end GPU.

We found empirically that the pre-processing stage should be computed in double-precision arithmetic whereas the real-time stage can be performed in single-precision arithmetic for accommodating more modes inside the GPU and a largerROI(i.e., the whole brain/head). A GPU with only 4 GB of dedicated memory is large enough for this process accommodating up to 400 modes, pre-computed primary fields, Huygens’s surface nodes, and anROIof size 220,000 samples on the middle grey matter surface.

Future work includes connecting this GPU-based E-field computation and GPU-based visualization within one screen refresh in real-time. The integration of real-time E-field modeling in combination with neuronavigation and TMS stimulator control will enable several avenues of research and clinical intervention. For example, this methodology will enable support for more reliable dosing throughout a TMS session by automatically changing TMS intensities accounting for coil placement variability. It will also allow the development of methods for faster motor threshold determination applying fewer TMS pulses by using E-field-based instead of grid search approaches. Furthermore, this approach will allow the development of rapid coil placement optimization for updated brain targets and E-field constraints during the TMS procedure. Updated brain targeting may be derived from behavioral (e.g., task performance, or treatment indicators) or physiological (e.g., heart rate variability, or electroencephalography) response even in closed-loop settings.

## 5 Conclusions

We have developed a method for rapidly calculating the TMS-induced E-field. This method is based on a functional generalization of the probabilistic matrix decomposition and generalized Huygens’s and reciprocity principles. The initial preprocessing stage takes approximately 40 hours to complete given the algorithm requires solving 400 FEM simulations and 400 ADM simulations. However, subsequent E-field computations can be done within 4 ms in a GPU and 1.2 s in a CPU over the middle grey matter surface (containing, on average, 216,000 barycentric points). The resultant E-field has accuracy comparable to standard FEM solutions. Notably, this computational performance can be achieved using a standard GPU with a dedicated memory of only 4 GB, making it practical for many users. This framework enables real-time E-field calculation in the cortex for arbitrary coil design and coil placement, and generalizes well for distinct head model pipelines, underscoring its adaptability and suitability for a wide range of applications.

## Supplementary Material

Supplementary Material

## Data Availability

In-house codes are available athttps://github.com/NahianHasan/Real-Time-TMS

## Appendix

**Appendix Table 1.** Abbreviation and notation.

Notation	Definition
$N_{m}$	Number of modes
$N_{e}$	Number of brain tetrahedrons
$N_{d}$	Number of triangular facets in the Huygens’s surface mesh
$I^{\prime }\left(t\right)$	Time derivative of the driving current pulse waveform during TMS
${\text{W}}^{\left(i\right)}({\text{r}}_{j})$	$i^{\text{th}}$ sample of white noise current at centers of $j^{\text{th}}$ triangular facet ( $j\in \left\{1,2,...,\;N_{d}\right\}$ ) in the Huygens’s surface; $i\in \left\{1,2,...,N_{m}\right\}$
${\text{E}}_{\text{W}}^{\text{P}\left(i\right)}\left(\text{r}\right)$	Primary E-field induced in the brain by current source ${\text{W}}^{\left(i\right)}({\text{r}}_{j})$ ; $i\in \left\{1,2,...,N_{m}\right\}$
$E_{\text{W}}^{\left(i\right)}\left(\text{r}\right)$	Total induced E-field in the brain generated by current source ${\text{W}}^{\left(i\right)}({\text{r}}_{j})$ ; ( $i\in \left\{1,2,...,N_{m}\right\}$ )
$\phi^{\left(i\right)}\left(\text{r}\right)$	Scalar potential in the brain; $i\in \left\{1,2,...,N_{m}\right\}$
${\text{M}}^{\left(i\right)}\left(\text{r}\right)$	$i^{\text{th}}$ orthonormal mode function
${\text{E}}_{\text{M}}^{\left(i\right)}\left(\text{r}\right)$ , ${\text{H}}_{\text{M}}^{\left(i\right)}\left(\text{r}\right)$	E-field and H-field generated by $i^{\text{th}}$ impressed current (mode function) ${\text{M}}^{\left(i\right)}\left(\text{r}\right)$ ; $i\in \left\{1,2,...,N_{m}\right\}$
${\text{J}}_{\text{S}}^{\left(i\right)}\left(\text{r}\right)$ , ${\text{K}}_{S}^{\left(i\right)}\left(\text{r}\right)$	Electric and magnetic current on the Huygens’s surface, S , for $i^{\text{th}}$ mode function; $i\in \left\{1,2,...,N_{m}\right\}$
${\text{E}}_{{\text{J}}_{S},{\text{K}}_{S}}^{\left(i\right)}\left(\text{r}\right)$	E-field generated by ${\text{J}}_{S}^{\left(i\right)}\left(\text{r}\right)$ and ${\text{K}}_{S}^{\left(i\right)}\left(\text{r}\right)$ ; $i\in \left\{1,2,...,N_{m}\right\}$
${\text{E}}_{\text{TMS}}^{\text{P}}\left(\text{r}\right)$ , ${\text{H}}_{\text{TMS}}^{\text{P}}\left(\text{r}\right)$	Primary TMS E-field and H-field generated by any coil model
T	Transformation matrix for relative placement of Huygens’s surface with respect to the coil
$a^{\left(i\right)}$	Mode coefficient corresponding to $i^{\text{th}}$ mode function $\left({\text{M}}^{\left(i\right)}\left(\text{r}\right)\right)$ ; $i\in \left\{1,2,...,N_{m}\right\}$
${\text{J}}_{\text{TMS}}\left(\text{r}\right)$	Electric current density distribution on the TMS coil
${\text{E}}_{\text{TMS}}\left(\text{r}\right)$	Actual TMS induced E-field
${\text{E}}_{\text{TMS}}^{\left(N_{m}\right)}\left(\text{r}\right)$	Real-time approximated TMS induced E-field from $N_{m}$ mode functions
$A_{j}$	Area of the $j^{\text{th}}$ triangular facet in the Huygens’s surface; $j\in \left\{1,2,...,N_{d}\right\}$
$V_{k}$	Volume of the $k^{\text{th}}$ tetrahedron in the head mesh; $k\in \left\{1,2,...,N_{e}\right\}$
${\text{R}}_{c}$	3×3 rotation matrix for TMS coil with respect to the head
${\text{T}}_{0}$	Translation vector for the TMS coil placement with respect to the head
σ(r)	Conductivity at location r in the head
$\hat{\text{n}}$	Normal vector on the scalp surface pointing outward
S	Huygens’s surface
Ω	Brain region in the head

## References

[b1] Balanis , C. A. ( 2012 ). Advanced engineering electromagnetics . John Wiley & Sons . 10.1002/9781394180042

[b2] Barker , A. T. , Jalinous , R. , & Freeston , I. L. ( 1985 ). Non-invasive magnetic stimulation of human motor cortex . The Lancet , 325 ( 8437 ), 1106 – 1107 . 10.1016/s0140-6736(85)92413-4 2860322

[b3] Beynel , L. , Davis , S. W. , Crowell , C. A. , Dannhauer , M. , Lim , W. , Palmer , H. , Hilbig , S. A. , Brito , A. , Hile , C. , Luber , B. , Lisanby , S. H. , Peterchev , A. V. , Cabeza , R. , & Appelbaum , L. G. ( 2020 ). Site-specific effects of online rTMS during a working memory task in healthy older adults . Brain Sciences , 10 ( 5 ). 10.3390/brainsci10050255 PMC7287855 32349366

[b4] Carmi , L. , Tendler , A. , Bystritsky , A. , Hollander , E. , Blumberger , D. M. , Daskalakis , J. , Ward , H. , Lapidus , K. , Goodman , W. , Casuto , L. , Feifel , D. , Barnea-Ygael , N. , Roth , Y. , Zangen , A. , & Zohar , J. ( 2019 ). Efficacy and safety of deep transcranial magnetic stimulation for obsessive-compulsive disorder: A prospective multicenter randomized double-blind placebo-controlled trial . American Journal of Psychiatry , 176 ( 11 ), 931 – 938 . 10.1176/appi.ajp.2019.18101180 31109199

[b5] Cohen , S. L. , Bikson , M. , Badran , B. W. , & George , M. S. ( 2022 ). A visual and narrative timeline of US FDA milestones for transcranial magnetic stimulation (TMS) devices . Brain Stimulation: Basic, Translational, and Clinical Research in Neuromodulation , 15 ( 1 ), 73 – 75 . 10.1016/j.brs.2021.11.010 PMC8864803 34775141

[b6] Couturier , J. L. ( 2005 ). Efficacy of rapid-rate repetitive transcranial magnetic stimulation in the treatment of depression: A systematic review and meta-analysis . Journal of Psychiatry and Neuroscience , 30 ( 2 ), 83 – 90 . 10.1186/s12884-021-03600-3 15798783 PMC551158

[b7] Daneshzand , M. , de Lara , L. I. N. , Meng , Q. , Makarov , S. , Uluç , I. , Ahveninen , J. , Raij , T. , & Nummenmaa , A. ( 2023 ). Experimental verification of a computational real-time neuronavigation system for multichannel transcranial magnetic stimulation . Brain and Human Body Modelling , 2021 , 61 . 10.1007/978-3-031-15451-5_4

[b8] Daneshzand , M. , Makarov , S. N. , de Lara , L. I. N. , Guerin , B. , McNab , J. , Rosen , B. R. , Hämäläinen , M. S. , Raij , T. , & Nummenmaa , A. ( 2021 ). Rapid computation of TMS-induced E-fields using a dipole-based magnetic stimulation profile approach . Neuroimage , 237 , 118097 . 10.1016/j.neuroimage.2021.118097 33940151 PMC8353625

[b9] Dannhauer , M. , Huang , Z. , Beynel , L. , Wood , E. , Bukhari-Parlakturk , N. , & Peterchev , A. V. ( 2022 ). Tap: Targeting and analysis pipeline for optimization and verification of coil placement in transcranial magnetic stimulation . Journal of Neural Engineering , 19 ( 2 ), 026050 . 10.1088/1741-2552/ac63a4 PMC9131512 35377345

[b10] De Ridder , D. , De Mulder , G. , Menovsky , T. , Sunaert , S. , & Kovacs , S. ( 2007 ). Electrical stimulation of auditory and somatosensory cortices for treatment of tinnitus and pain . Progress in Brain Research , 166 , 377 – 388 . 10.1016/s0079-6123(07)66036-1 17956802

[b11] Deng , Z.-D. , Lisanby , S. H. , & Peterchev , A. V. ( 2013 ). Electric field depth–focality tradeoff in transcranial magnetic stimulation: Simulation comparison of 50 coil designs . Brain Stimulation , 6 ( 1 ), 1 – 13 . 10.1016/j.brs.2012.02.005 22483681 PMC3568257

[b12] Gomez , L. J. , Dannhauer , M. , Koponen , L. , & Peterchev , A. V. ( 2020a ). TMS_Efield_Solvers . https://github.com/luisgo/TMS_Efield_Solvers.git

[b13] Gomez , L. J. , Dannhauer , M. , Koponen , L. M. , & Peterchev , A. V. ( 2020b ). Conditions for numerically accurate TMS electric field simulation . Brain Stimulation , 13 ( 1 ), 157 – 166 . 10.1016/j.brs.2019.09.015 31604625 PMC6888902

[b14] Gomez , L. J. , Dannhauer , M. , & Peterchev , A. V. ( 2021 ). Fast computational optimization of TMS coil placement for individualized electric field targeting . Neuroimage , 228 , 117696 . 10.1016/j.neuroimage.2020.117696 33385544 PMC7956218

[b15] Gomez , L. J. , Goetz , S. M. , & Peterchev , A. V. ( 2018 ). Design of transcranial magnetic stimulation coils with optimal trade-off between depth, focality, and energy . Journal of Neural Engineering , 15 ( 4 ), 046033 . 10.1088/1741-2552/aac967 29855433 PMC6433395

[b16] Greengard , L. , & Gimbutas , Z. ( 2012 ). FMM3D Software . 10.4208/cicp.150215.260615sw

[b17] Hasan , N. I. , Wang , D. , & Gomez , L. J. ( 2023 ). Fast and accurate computational E-field dosimetry for group-level transcranial magnetic stimulation targeting . Computers in Biology and Medicine , 167 , 107614 . 10.1016/j.compbiomed.2023.107614 37913615 PMC10880124

[b18] Hoffman , R. E. , Gueorguieva , R. , Hawkins , K. A. , Varanko , M. , Boutros , N. N. , te Wu , Y., Carroll , K. , & Krystal , J. H. ( 2005 ). Temporoparietal transcranial magnetic stimulation for auditory hallucinations: Safety, efficacy and moderators in a fifty patient sample . Biological Psychiatry , 58 ( 2 ), 97 – 104 . 10.1016/j.biopsych.2005.03.041 15936729

[b19] Kaptsan , A. , Yaroslavsky , Y. , Applebaum , J. , Belmaker , R. H. , & Grisaru , N. ( 2003 ). Right prefrontal TMS versus sham treatment of mania: A controlled study . Bipolar Disorders , 5 ( 1 ), 36 – 39 . 10.1034/j.1399-5618.2003.00003.x 12656936

[b20] Kreyszig , E. ( 1972 ). Advanced engineering mathematics . John Wiley & Sons . 10.1002/bimj.19650070232

[b21] Laakso , I. , & Hirata , A. ( 2012 ). Fast multigrid-based computation of the induced electric field for transcranial magnetic stimulation . Physics in Medicine & Biology , 57 ( 23 ), 7753 . 10.1088/0031-9155/57/23/7753 23128377

[b22] Lan , L. , Zhang , X. , Li , X. , Rong , X. , & Peng , Y. ( 2017 ). The efficacy of transcranial magnetic stimulation on migraine: A meta-analysis of randomized controlled trails . The Journal of Headache and Pain , 18 ( 1 ), 1 – 7 . 10.1186/s10194-017-0792-4 28831756 PMC5567575

[b23] Lee , E. G. , Duffy , W. , Hadimani , R. L. , Waris , M. , Siddiqui , W. , Islam , F. , Rajamani , M. , Nathan , R. , & Jiles , D. C. ( 2016 ). Investigational effect of brain-scalp distance on the efficacy of transcranial magnetic stimulation treatment in depression . IEEE Transactions on Magnetics , 52 ( 7 ), 1 – 4 . 10.1109/tmag.2015.2514158

[b24] Lipton , R. B. , Dodick , D. W. , Silberstein , S. D. , Saper , J. R. , Aurora , S. K. , Pearlman , S. H. , Fischell , R. E. , Ruppel , P. L. , & Goadsby , P. J. ( 2010 ). Single-pulse transcranial magnetic stimulation for acute treatment of migraine with aura: A randomised, double-blind, parallel-group, sham-controlled trial . The Lancet Neurology , 9 ( 4 ), 373 – 380 . 10.1016/s1474-4422(10)70054-5 20206581

[b25] Makarov , S. N. ( 2020 ). TMS-Base-Package-040120 . https://github.com/TMSCoreLab/TMS-Base-Package-040120

[b26] Makarov , S. N. , Wartman , W. A. , Daneshzand , M. , Fujimoto , K. , Raij , T. , & Nummenmaa , A. ( 2020 ). A software toolkit for TMS electric-field modeling with boundary element fast multipole method: An efficient Matlab implementation . Journal of Neural Engineering , 17 ( 4 ), 046023 . 10.1088/1741-2552/ab85b3 32235065

[b27] MATLAB . ( 2022 ). version 9.8.0 (R2020a) . The MathWorks Inc. , Natick, MA . 10.1177/106480460301100306

[b28] Nahas , Z. , Kozel , F. A. , Li , X. , Anderson , B. , & George , M. S. ( 2003 ). Left prefrontal transcranial magnetic stimulation (TMS) treatment of depression in bipolar affective disorder: A pilot study of acute safety and efficacy . Bipolar Disorders , 5 ( 1 ), 40 – 47 . 10.1034/j.1399-5618.2003.00011.x 12656937

[b29] Nielsen , J. D. , Madsen , K. H. , Puonti , O. , Siebner , H. R. , Bauer , C. , Madsen , C. G. , Saturnino , G. B. , & Thielscher , A. ( 2018 ). Automatic skull segmentation from MR images for realistic volume conductor models of the head: Assessment of the state-of-the-art . Neuroimage , 174 , 587 – 598 . 10.1016/j.neuroimage.2018.03.001 29518567

[b30] Nieminen , A. E. , Nieminen , J. O. , Stenroos , M. , Novikov , P. , Nazarova , M. , Vaalto , S. , Nikulin , V. , & Ilmoniemi , R. J. ( 2022 ). Accuracy and precision of navigated transcranial magnetic stimulation . Journal of Neural Engineering , 19 ( 6 ), 066037 . 10.1088/1741-2552/aca71a 36541458

[b31] Nummenmaa , A. , Stenroos , M. , Ilmoniemi , R. J. , Okada , Y. C. , Hämäläinen , M. S. , & Raij , T. ( 2013 ). Comparison of spherical and realistically shaped boundary element head models for transcranial magnetic stimulation navigation . Clinical Neurophysiology , 124 ( 10 ), 1995 – 2007 . 10.1016/j.clinph.2013.04.019 23890512 PMC3790855

[b32] O’Reardon , J. P. , Solvason , H. B. , Janicak , P. G. , Sampson , S. , Isenberg , K. E. , Nahas , Z. , McDonald , W. M. , Avery , D. , Fitzgerald , P. B. , Loo , C. , Demitrack , M. A. , George , M. S. , & Sackeim , H. A. ( 2007 ). Efficacy and safety of transcranial magnetic stimulation in the acute treatment of major depression: A multisite randomized controlled trial . Biological Psychiatry , 62 ( 11 ), 1208 – 1216 . 10.1016/j.biopsych.2007.01.018 17573044

[b33] Pascual-Leone , A. , Rubio , B. , Pallardó , F. , & Catalá , M. D. ( 1996 ). Rapid-rate transcranial magnetic stimulation of left dorsolateral prefrontal cortex in drug-resistant depression . The Lancet , 348 ( 9022 ), 233 – 237 . 10.1016/s0140-6736(96)01219-6 8684201

[b34] Paulus , W. , Peterchev , A. V. , & Ridding , M. ( 2013 ). Transcranial electric and magnetic stimulation: Technique and paradigms . Handbook of Clinical Neurology , 116 , 329 – 342 . 10.1016/b978-0-444-53497-2.00027-9 24112906

[b35] Peterson , A. F. , Ray , S. L. , & Mittra , R. ( 1998 ). Computational methods for electromagnetics , volume 351. IEEE Press , New York . 10.1109/9780470544303

[b36] Plonsey , R. ( 1972 ). Capability and limitations of electrocardiography and magnetocardiography . IEEE Transactions on Biomedical Engineering , 19 ( 3 ), 239 – 244 . 10.1109/tbme.1972.324123 5021223

[b37] Sollmann , N. , Goblirsch-Kolb , M. F. , Ille , S. , Butenschoen , V. M. , Boeckh-Behrens , T. , Meyer , B. , Ringel , F. , & Krieg , S. M. ( 2016 ). Comparison between electric-field-navigated and line-navigated TMS for cortical motor mapping in patients with brain tumors . Acta Neurochirurgica , 158 , 2277 – 2289 . 10.1007/s00701-016-2970-6 27722947

[b38] Stenroos , M. , & Koponen , L. M. ( 2019 ). Real-time computation of the TMS-induced electric field in a realistic head model . NeuroImage , 203 , 116159 . 10.1016/j.neuroimage.2019.116159 31494248

[b39] Stenroos , M. , Mylläri , T. , & Jyrkinen , K. ( 2023 ). GPU-accelerated solutions to forward problem of TMS . Brain Stimulation , 16 ( 1 ), 395 – 396 . 10.1016/j.brs.2023.01.797

[b40] Tai , C.-T. ( 1998 ). Direct integration of field equations . In IEEE Antennas and Propagation Society International Symposium , Vol. 2 , p. 884 . 10.1109/aps.1998.702084

[b41] Thielscher , A. , Antunes , A. , & Saturnino , G. B. ( 2015 ). Field modeling for transcranial magnetic stimulation: A useful tool to understand the physiological effects of TMS. In 2015 37th Annual International Conference of the IEEE Engineering in Medicine and Biology Society (EMBC) , pp. 222 – 225 . IEEE . 10.1109/embc.2015.7318340 26736240

[b42] Wagner , T. A. , Zahn , M. , Grodzinsky , A. J. , & Pascual-Leone , A. ( 2004 ). Three-dimensional head model simulation of transcranial magnetic stimulation . IEEE Transactions on Biomedical Engineering , 51 ( 9 ), 1586 – 1598 . 10.1109/tbme.2004.827925 15376507

[b43] Wang , B. , Peterchev , A. V. , Gaugain , G. , Ilmoniemi , R. J. , Grill , W. M. , Bikson , M. , & Nikolayev , D. ( 2024 ). Quasistatic approximation in neuromodulation . arXiv preprint arXiv:2402.00486 . 10.1088/1741-2552/ad625e PMC11370654 38994790

[b44] Wang , D. , Hasan , N. I. , Dannhauer , M. , Yucel , A. C. , & Gomez , L. J. ( 2023 ). Fast computational E-field dosimetry for transcranial magnetic stimulation using adaptive cross approximation and auxiliary dipole method (ACA-ADM) . NeuroImage , 267 , 119850 . 10.1016/j.neuroimage.2022.119850 36603745 PMC11658687

[b45] Weise , K. , Numssen , O. , Kalloch , B. , Zier , A. L. , Thielscher , A. , Haueisen , J. , Hartwigsen , G. , & Knösche , T. R. ( 2023 ). Precise motor mapping with transcranial magnetic stimulation . Nature Protocols , 18 ( 2 ), 293 – 318 . 10.1038/s41596-022-00776-6 36460808

[b46] Windhoff , M. , Opitz , A. , & Thielscher , A. ( 2013 ). Electric field calculations in brain stimulation based on finite elements: An optimized processing pipeline for the generation and usage of accurate individual head models . Technical Report . Wiley Online Library . 10.1002/hbm.21479 PMC6870291 22109746

[b47] Xu , G. , Rathi , Y. , Camprodon , J. A. , Cao , H. , & Ning , L. ( 2021 ). Rapid whole-brain electric field mapping in transcranial magnetic stimulation using deep learning . PLoS One , 16 ( 7 ), e0254588 . 10.1371/journal.pone.0254588 34329328 PMC8323956

[b48] Yokota , T. , Maki , T. , Nagata , T. , Murakami , T. , Ugawa , Y. , Laakso , I. , Hirata , A. , & Hontani , H. ( 2019 ). Real-time estimation of electric fields induced by transcranial magnetic stimulation with deep neural networks . Brain Stimulation , 12 ( 6 ), 1500 – 1507 . 10.1016/j.brs.2019.06.015 31262697

