# Supplementary material for: Real-time computation of brain E-field for enhanced transcranial magnetic stimulation neuronavigation and optimization
Source: Imaging Neurosci (Camb). 2025 Jan 2;3:imag_a_00412. doi: 10.1162/imag_a_00412 (PMC12319877; doi:10.1162/imag_a_00412)
Supplement: Supplementary Material [file imag_a_00412-supp.pdf]

6. Supplementary Material

6.1. Temporal and spatial variation of fields due to source outside the head

Assume the TMS coil has an electric current density of $\mathbf{J}_{\mathbf{TMS}}(\mathbf{r};t)$ that generates an E-field $\mathbf{E}_{\mathbf{TMS}}(\mathbf{r};t)$ inside the head with a conductivity distribution of $\sigma(\mathbf{r})$. Since the TMS current pulse has a relatively long rise time relative to the speed of light, quasi-static assumptions are valid. We can neglect the displacement currents. This results in the following equations for the H-field ($\mathbf{H}_{\mathbf{TMS}}(\mathbf{r};t)$)

$$\begin{aligned} \nabla \times \mathbf{H}_{\mathbf{TMS}}(\mathbf{r};t) &= \sigma(\mathbf{r})\mathbf{E}_{\mathbf{TMS}}(\mathbf{r};t) + \mathbf{J}_{\mathbf{TMS}}(\mathbf{r};t), \\ \nabla \cdot \mu_0 \mathbf{H}_{\mathbf{TMS}}(\mathbf{r};t) &= 0, \end{aligned} \tag{S1}$$

where μ_0 is the magnetic permeability. In this case, the H-field is purely rotational, allowing it to be expressed as $\mu_0\mathbf{H}_{\mathbf{TMS}}(\mathbf{r};t) = \nabla \times \mathbf{A}_{\mathbf{TMS}}(\mathbf{r};t)$, where $\mathbf{A}_{\mathbf{TMS}}(\mathbf{r};t)$ is the vector potential. Now, if we assume coulomb gauge (i.e., $\nabla \cdot \mathbf{A}_{\mathbf{TMS}}(\mathbf{r};t) = 0$), Equation (S1) can be expressed as

$$\begin{aligned} \frac{1}{\mu_0}\nabla \times \nabla \times \mathbf{A}_{\mathbf{TMS}}(\mathbf{r};t) &= \frac{1}{\mu_0}\nabla(\nabla \cdot \mathbf{A}_{\mathbf{TMS}}(\mathbf{r};t)) - \frac{1}{\mu_0}\nabla^2\mathbf{A}_{\mathbf{TMS}}(\mathbf{r};t), \\ &= -\frac{1}{\mu_0}\nabla^2\mathbf{A}_{\mathbf{TMS}}(\mathbf{r};t), \\ &= \sigma(\mathbf{r})\mathbf{E}_{\mathbf{TMS}}(\mathbf{r};t) + \mathbf{J}_{\mathbf{TMS}}(\mathbf{r};t). \end{aligned} \tag{S2}$$

Equation (S2) can be solved for $\mathbf{A}_{\mathbf{TMS}}(\mathbf{r};t)$ using a Green's function as

$$\mathbf{A}_{\mathbf{TMS}}(\mathbf{r};t) = \frac{\mu_0}{4\pi}\int_{\mathbb{R}^3} \frac{\sigma(\mathbf{r}')\mathbf{E}_{\mathbf{TMS}}(\mathbf{r}';t) + \mathbf{J}_{\mathbf{TMS}}(\mathbf{r}';t)}{||\mathbf{r}-\mathbf{r}'||}\,\mathrm{d}\mathbf{r}'. \tag{S3}$$

Typically, the mutual inductive coupling is small (i.e. the body negligibly distorts the magnetic field), as such,

$$\mathbf{A}_{\mathbf{TMS}}(\mathbf{r};t) \approx \frac{\mu_0}{4\pi}\int_{\mathbb{R}^3} \frac{\mathbf{J}_{\mathbf{TMS}}(\mathbf{r}';t)}{||\mathbf{r}-\mathbf{r}'||}\,\mathrm{d}\mathbf{r}' = \frac{\mu_0}{4\pi}\mathrm{I}(t)\int_{\mathbb{R}^3} \frac{\mathbf{J}_{\mathbf{TMS}}(\mathbf{r}')}{||\mathbf{r}-\mathbf{r}'||}\,\mathrm{d}\mathbf{r}'. \tag{S4}$$

Therefore,

$$\mathbf{H}_{\mathbf{TMS}}(\mathbf{r};t) = \frac{1}{\mu_0}\mathrm{I}(t)\nabla \times \mathbf{A}_{\mathbf{TMS}}(\mathbf{r}), \tag{S5}$$

where

$$\mathbf{A}_{\mathbf{TMS}}(\mathbf{r}) = \frac{\mu_0}{4\pi}\int_{\mathbb{R}^3} \frac{\mathbf{J}_{\mathbf{TMS}}(\mathbf{r}')}{||\mathbf{r}-\mathbf{r}'||}\,\mathrm{d}\mathbf{r}'. \tag{S6}$$

In other words, the temporal variation is separable. On the other hand, according to Faraday's law, we can express $\mathbf{E}_{\mathbf{TMS}}(\mathbf{r};t)$ as

$$\nabla \times \mathbf{E}_{\mathbf{TMS}}(\mathbf{r};t) = -\mu_0\frac{\mathrm{d}}{\mathrm{d}t}\mathbf{H}_{\mathbf{TMS}}(\mathbf{r};t). \tag{S7}$$

Replacing $\mu_0\mathbf{H}_{\mathbf{TMS}}(\mathbf{r};t)$ with $\nabla \times \mathbf{A}_{\mathbf{TMS}}(\mathbf{r};t)$ and rearranging, we have

$$\nabla \times \left(\mathbf{E}_{\mathbf{TMS}}(\mathbf{r};t) + \frac{\mathrm{d}}{\mathrm{d}t}\mathbf{A}_{\mathbf{TMS}}(\mathbf{r};t)\right) = 0. \tag{S8}$$

As such, combining the rotational part ($\frac{\mathrm{d}}{\mathrm{d}t}\mathbf{A}_{\mathbf{TMS}}(\mathbf{r};t)$) and irrotational part (- gradient of scalar potential, $\phi_{\mathrm{TMS}}(\mathbf{r};t)$), $\mathbf{E}_{\mathbf{TMS}}(\mathbf{r};t)$ can be expressed as

$$\mathbf{E}_{\mathbf{TMS}}(\mathbf{r};t) = -\frac{\mathrm{d}}{\mathrm{d}t}\mathbf{A}_{\mathbf{TMS}}(\mathbf{r};t) - \nabla\phi_{\mathrm{TMS}}(\mathbf{r};t). \tag{S9}$$

Now, taking the divergence of Equation (S1) and replacing $\mathbf{E}_{\mathbf{TMS}}(\mathbf{r};t)$ with Equation (S9), we have

$$\nabla \cdot \left[\sigma(\mathbf{r})\left(-\frac{\mathrm{d}}{\mathrm{d}t}\mathbf{A}_{\mathbf{TMS}}(\mathbf{r};t) - \nabla\phi_{\mathrm{TMS}}(\mathbf{r};t)\right) + \mathbf{J}_{\mathbf{TMS}}(\mathbf{r};t)\right] = 0. \tag{S10}$$

Note that the coil current density is divergence-free, in other words, $\nabla \cdot \mathbf{J}_{\mathbf{TMS}}(\mathbf{r};t) = 0$. We can also assume $\phi_{\mathrm{TMS}}(\mathbf{r};t) = \mathrm{I}'(t)\phi_{\mathrm{TMS}}(\mathbf{r})$, where $\mathrm{I}'(t) = \frac{\mathrm{d}}{\mathrm{d}t}\mathrm{I}(t)$. Consequently, the temporal derivative of

the pulse-driving current cancels from both sides. Rearranging Equation (S10) and using Equation (S4), we get

$$-\nabla \cdot \sigma(\mathbf{r})\nabla\phi_{\text{TMS}}(\mathbf{r}) = \mu_0\nabla \cdot \sigma(\mathbf{r})\mathbf{A}_{\mathbf{TMS}}(\mathbf{r}). \tag{S11}$$

We use Equation (S11) to solve for the scalar potential inside the head (as shown in Equation (4)) for randomly distributed magnetic current distributions. However, Equation (S9) can now be expressed as

$$\begin{aligned} \mathbf{E}_{\mathbf{TMS}}(\mathbf{r};t) &= \text{I}'(t)\Big(-\mu_0\mathbf{A}_{\mathbf{TMS}}(\mathbf{r}) - \nabla\phi_{\text{TMS}}(\mathbf{r})\Big), \\ &= \text{I}'(t)\mathbf{E}_{\mathbf{TMS}}(\mathbf{r}), \end{aligned} \tag{S12}$$

where $\mathbf{E}_{\mathbf{TMS}}(\mathbf{r}) = -\mu_0\mathbf{A}_{\mathbf{TMS}}(\mathbf{r}) - \nabla\phi_{\text{TMS}}(\mathbf{r})$.

In summary, due to the quasi-static nature of the fields, the temporal and spatial variations of the E-field and H-field can be separated. As is evident from Equation (S5) and (S12), the H-field varies as $\text{I}(t)$ and the E-field varies as $\text{I}'(t)$, respectively.

6.2. Temporal and spatial variation of fields due to impressed current sources inside the head

Unlike the previous section, in this section, we assume the current sources are residing inside the head. Assume, there are impressed current sources $\mathbf{M}(\mathbf{r};t)$ inside the head. Due to the quasi-static nature of TMS, $\mathbf{M}(\mathbf{r};t) = \text{I}(t)\mathbf{M}(\mathbf{r})$. Using Ampere's law and neglecting displacement currents as before, we can relate the resultant H-field ($\mathbf{H}_{\mathbf{M}}(\mathbf{r};t)$), the E-field ($\mathbf{E}_{\mathbf{M}}(\mathbf{r};t)$) and sources ($\mathbf{M}(\mathbf{r};t)$) as follows-

$$\nabla \times \mathbf{H}_{\mathbf{M}}(\mathbf{r};t) = \sigma(\mathbf{r})\mathbf{E}_{\mathbf{M}}(\mathbf{r};t) + \mathbf{M}(\mathbf{r};t). \tag{S13}$$

As before, $\mathbf{H_M}(\mathbf{r};t)$ is purely rotational, as such, $\mu_0\mathbf{H_M}(\mathbf{r};t) = \nabla\times\mathbf{A_M}(\mathbf{r};t)$, where $\mathbf{A_M}(\mathbf{r};t)$ is the magnetic vector potential. Assuming coulomb gauge (i.e., $\nabla\cdot\mathbf{A_M}(\mathbf{r};t) = 0$), it can be shown that

$$\frac{1}{\mu_0}\nabla\times\nabla\times\mathbf{A_M}(\mathbf{r};t) = -\frac{1}{\mu_0}\nabla^2\mathbf{A_M}(\mathbf{r};t) \quad = \sigma(\mathbf{r})\mathbf{E_M}(\mathbf{r};t) + \mathbf{M}(\mathbf{r};t). \tag{S14}$$

Equation (S14) can be solved using Green's function inside the head (since $\sigma(\mathbf{r})$ and $\mathbf{M}(\mathbf{r};t)$ are only non-zero inside the head) as

$$\mathbf{A_M}(\mathbf{r};t) = \frac{\mu_0}{4\pi}\int_{Head}\frac{\sigma(\mathbf{r}')\mathbf{E_M}(\mathbf{r}';t) + \mathbf{M}(\mathbf{r}';t)}{||\mathbf{r}-\mathbf{r}'||}\,\mathrm{d}\mathbf{r}'. \tag{S15}$$

Again, we can express the total E-field as (similar to Equation (S9))

$$\mathbf{E_M}(\mathbf{r};t) = -\frac{\mathrm{d}}{\mathrm{d}t}\mathbf{A_M}(\mathbf{r};t) - \nabla\phi_{\mathrm{M}}(\mathbf{r};t), \tag{S16}$$

where $\phi_{\mathrm{M}}(\mathbf{r};t)$ is the scalar potential. Taking the divergence of Equation (S13) results in

$$\nabla\cdot\left[\sigma(\mathbf{r})\left(-\frac{\mathrm{d}}{\mathrm{d}t}\mathbf{A_M}(\mathbf{r};t) - \nabla\phi_{\mathrm{M}}(\mathbf{r};t)\right) + \mathbf{M}(\mathbf{r};t)\right] = 0. \tag{S17}$$

Unlike in section (6.1), the sources here are not divergence-free (i.e., $\nabla\cdot\mathbf{M}(\mathbf{r};t)\neq 0$).

Inside the head, according to quasi-static assumptions, $\left\|\frac{\mathrm{d}}{\mathrm{d}t}\mathbf{A_M}(\mathbf{r};t)\right\| << \left\|\nabla\phi_{\mathrm{M}}(\mathbf{r};t)\right\|$. As a result, inside the head $\mathbf{E_M}(\mathbf{r};t)\approx -\nabla\phi_{\mathrm{M}}(\mathbf{r};t)$ and $-\nabla\cdot\mathbf{M}(\mathbf{r};t)\approx -\nabla\cdot\sigma(\mathbf{r})\nabla\phi_{\mathrm{M}}(\mathbf{r};t)$. To determine $\phi_{\mathrm{M}}(\mathbf{r};t)$, we solve equation (S17) using standard nodal elements. Furthermore, since the scalar potential is generated by $\mathrm{M}(\mathbf{r};t) = \mathrm{I}(t)\mathrm{M}(\mathbf{r})$, we can assume, $\phi_{\mathrm{M}}(\mathbf{r};t) = \mathrm{I}(t)\phi_{\mathrm{M}}(\mathbf{r})$. Once we know the scalar potential inside the head, we can determine the vector potential outside of the head by the following equation

$$\begin{aligned}\mathbf{A_M}(\mathbf{r};t) &= \frac{\mu_0}{4\pi}\mathrm{I}(t)\int_{Head}\frac{\sigma(\mathbf{r}')\nabla\phi_M(\mathbf{r}') + \mathbf{M}(\mathbf{r}')}{||\mathbf{r}-\mathbf{r}'||}\,\mathrm{d}\mathbf{r}', \\ &= \mathrm{I}(t)\mathbf{A_M}(\mathbf{r}),\end{aligned} \tag{S18}$$

where $\mathbf{A_M}(\mathbf{r}) = \frac{\mu_0}{4\pi}\int_{Head}\frac{\sigma(\mathbf{r}')\nabla\phi_M(\mathbf{r}')+\mathbf{M}(\mathbf{r}')}{||\mathbf{r}-\mathbf{r}'||}\,\mathrm{d}\mathbf{r}'$. Consequently, $\mathbf{H_M}(\mathbf{r};t) = \frac{1}{\mu_0}\mathrm{I}(t)\nabla \times \mathbf{A_M}(\mathbf{r})$. Furthermore, the E-field satisfies

$$\mathbf{E_M}(\mathbf{r};t) \approx -\frac{\mathrm{d}}{\mathrm{d}t}\mathbf{A_M}(\mathbf{r};t) = -\mathrm{I}'(t)\mathbf{A_M}(\mathbf{r}). \tag{S19}$$

In summary, the H-field varies as $\mathrm{I}(t)$, whereas the E-field varies as $\mathrm{I}'(t)$ outside the head and as $\mathrm{I}(t)$ inside the head.

6.3. Electromagnetic reciprocity principle

The reciprocity principle establishes an equivalence relationship between two scenarios. In one scenario, the impressed electric current density $\mathbf{J_{TMS}}(\mathbf{r};t)$ generate an E-field $\mathbf{E_{TMS}}(\mathbf{r};t)$ and H-field $\mathbf{H_{TMS}}(\mathbf{r};t)$. Faraday's and Ampere's law dictate that

$$\nabla \times \mathbf{E_{TMS}}(\mathbf{r};t) = -\mu_0\frac{\mathrm{d}}{\mathrm{d}t}\mathbf{H_{TMS}}(\mathbf{r};t), \tag{S20a}$$

$$\nabla \times \mathbf{H_{TMS}}(\mathbf{r};t) = \sigma(\mathbf{r})\mathbf{E_{TMS}}(\mathbf{r};t) + \mathbf{J_{TMS}}(\mathbf{r};t), \tag{S20b}$$

respectively, where μ_0 is the magnetic permeability and $\sigma(\mathbf{r})$ is the conductivity distribution of the medium. In the second scenario, the electric current density sources $\mathbf{M}(\mathbf{r};t)$ inside the head induces an E-field $\mathbf{E_M}(\mathbf{r};t)$ and H-field $\mathbf{H_M}(\mathbf{r};t)$. As before, the corresponding relationships are

$$\nabla \times \mathbf{E_M}(\mathbf{r};t) = -\mu_0\frac{\mathrm{d}}{\mathrm{d}t}\mathbf{H_M}(\mathbf{r};t), \tag{S21a}$$

$$\nabla \times \mathbf{H_M}(\mathbf{r};t) = \sigma(\mathbf{r})\mathbf{E_M}(\mathbf{r};t) + \mathbf{M}(\mathbf{r};t). \tag{S21b}$$

In both cases, we have assumed constant μ_0, the absence of magnetic current density, and neglected the displacement current. Equation (S21b) dotted with $\mathbf{E_{TMS}}(\mathbf{r};t)$ is subtracted from Equation (S20a) dotted with $\mathbf{H_M}(\mathbf{r};t)$ resulting in

$$\begin{aligned} \mathbf{H_M}(\mathbf{r};t)\cdot\nabla\times\mathbf{E_{TMS}}(\mathbf{r};t) - \mathbf{E_{TMS}}(\mathbf{r};t)\cdot\nabla\times\mathbf{H_M}(\mathbf{r};t) = \nabla\cdot\big(\mathbf{E_{TMS}}(\mathbf{r};t)\times\mathbf{H_M}(\mathbf{r};t)\big) = \\ -\mu_0\mathbf{H_M}(\mathbf{r};t)\cdot\frac{\mathrm{d}}{\mathrm{d}t}\mathbf{H_{TMS}}(\mathbf{r};t) - \mathbf{E_{TMS}}(\mathbf{r};t)\cdot\sigma(\mathbf{r})\mathbf{E_M}(\mathbf{r};t) - \mathbf{E_{TMS}}(\mathbf{r};t)\cdot\mathbf{M}(\mathbf{r};t). \end{aligned} \tag{S22}$$

Using the vector identity $\nabla\cdot(\mathbf{A}\times\mathbf{B}) = \mathbf{B}\cdot(\nabla\times\mathbf{A}) - \mathbf{A}\cdot(\nabla\times\mathbf{B})$, the left hand side of Equation (S22) becomes $\nabla\cdot\big(\mathbf{E_{TMS}}(\mathbf{r};t)\times\mathbf{H_M}(\mathbf{r};t)\big)$. In a similar manner, Equation (S20b) dotted with $\mathbf{E_M}(\mathbf{r};t)$ is subtracted from Equation (S21a) dotted with $\mathbf{H_{TMS}}(\mathbf{r};t)$ resulting in

$$\begin{aligned} -\mathbf{E_M}(\mathbf{r};t)\cdot\nabla\times\mathbf{H_{TMS}}(\mathbf{r};t) + \mathbf{H_{TMS}}(\mathbf{r};t)\cdot\nabla\times\mathbf{E_M}(\mathbf{r};t) = \nabla\cdot\big(\mathbf{E_M}(\mathbf{r};t)\times\mathbf{H_{TMS}}(\mathbf{r};t)\big) = \\ -\mathbf{E_M}(\mathbf{r};t)\cdot\sigma(\mathbf{r})\mathbf{E_{TMS}}(\mathbf{r};t) - \mathbf{E_M}(\mathbf{r};t)\cdot\mathbf{J_{TMS}}(\mathbf{r};t) - \mu_0\mathbf{H_{TMS}}(\mathbf{r};t)\cdot\frac{\mathrm{d}}{\mathrm{d}t}\mathbf{H_M}(\mathbf{r};t). \end{aligned} \tag{S23}$$

Next, by subtracting Equation (S22) from (S23), we get

$$\nabla\cdot\big(\mathbf{E_M}(\mathbf{r};t)\times\mathbf{H_{TMS}}(\mathbf{r};t) - \mathbf{E_{TMS}}(\mathbf{r};t)\times\mathbf{H_M}(\mathbf{r};t)\big) = -\mathbf{E_M}(\mathbf{r};t)\cdot\mathbf{J_{TMS}}(\mathbf{r};t) + \mathbf{E_{TMS}}(\mathbf{r};t)\cdot\mathbf{M}(\mathbf{r};t). \tag{S24}$$

Integrating Equation (S24) over the volume Ω with the boundary $\partial\Omega$ and applying Gauss's law, we have

$$\begin{aligned} \int_{\partial\Omega}\big(\mathbf{E_M}(\mathbf{r};t)\times\mathbf{H_{TMS}}(\mathbf{r};t) - \mathbf{E_{TMS}}(\mathbf{r};t)\times\mathbf{H_M}(\mathbf{r};t)\big).\mathrm{d}\mathbf{r} = \\ \int_{\Omega}\big(-\mathbf{E_M}(\mathbf{r};t)\cdot\mathbf{J_{TMS}}(\mathbf{r};t) + \mathbf{E_{TMS}}(\mathbf{r};t)\cdot\mathbf{M}(\mathbf{r};t)\big)\mathrm{d}\mathbf{r}. \end{aligned} \tag{S25}$$

Let's assume the sources $\mathbf{J_{TMS}}(\mathbf{r};t)$ and $\mathbf{M}(\mathbf{r};t)$ can be enclosed by finite-sized small sphere. Then for an infinite-sized sphere, $\mathbf{H_M}(\mathbf{r};t) = \hat{\mathbf{r}}\times\frac{\mathbf{E_M}(\mathbf{r};t)}{\eta}$, $\mathbf{H_{TMS}}(\mathbf{r};t) = \hat{\mathbf{r}}\times\frac{\mathbf{E_{TMS}}(\mathbf{r};t)}{\eta}$, $\mathbf{E_M}(\mathbf{r};t)\cdot\hat{\mathbf{r}} = \mathbf{H_M}(\mathbf{r};t)\cdot\hat{\mathbf{r}} = \mathbf{H_{TMS}}(\mathbf{r};t)\cdot\hat{\mathbf{r}} = \mathbf{E_{TMS}}(\mathbf{r};t)\cdot\hat{\mathbf{r}} = 0$. Here, η is the free-space impedance

(Kong, 1986). With these source definitions and the vector identity $\mathbf{A}\times(\mathbf{B}\times\mathbf{C}) = \mathbf{B}(\mathbf{A}\cdot\mathbf{C})-\mathbf{C}(\mathbf{A}\cdot\mathbf{B})$, the left-hand side of Equation (S25) is evaluated to zero. Consequently, Equation (S25) takes the form

$$\int_{\Omega} \left(\mathbf{E_{TMS}}(\mathbf{r};t) \cdot \mathbf{M}(\mathbf{r};t) - \mathbf{E_M}(\mathbf{r};t) \cdot \mathbf{J_{TMS}}(\mathbf{r};t)\right) \mathrm{d}\mathbf{r} = 0. \tag{S26}$$

Now, $\mathbf{J_{TMS}}(\mathbf{r};t)$ is defined outside the head at coil location, whereas $\mathbf{M}(\mathbf{r};t)$ is defined inside the head. Also, the fields are quasi-static. Separating the temporal variations, the final form of reciprocity is

$$\mathrm{I}'(t)\mathrm{I}(t) \int_{\Omega} \mathbf{E_{TMS}}(\mathbf{r}) \cdot \mathbf{M}(\mathbf{r}) \mathrm{d}\mathbf{r} = \mathrm{I}'(t)\mathrm{I}(t) \int_{Coil} \mathbf{E_M}(\mathbf{r}) \cdot \mathbf{J_{TMS}}(\mathbf{r}) \mathrm{d}\mathbf{r}. \tag{S27}$$

The reciprocity form in Equation (S27) can be directly related to Equation (8).

6.4. Surface equivalent currents in Huygens's principle

According to the scalar-vector Green's theorem (detailed proof can be found in the Appendix of Tai (1998)),

$$\int_{\mathbf{\Omega}} \left[(\nabla \cdot \mathbf{P})\nabla p + \mathbf{P}\nabla \cdot \nabla p + f \nabla \times \nabla \times \mathbf{P}\right] \mathrm{d}\mathbf{r} = \int_{\partial\mathbf{\Omega}} \left[(\hat{\mathbf{n}} \cdot \mathbf{P})\nabla p + (\hat{\mathbf{n}} \times \mathbf{P}) \times \nabla p + (\hat{\mathbf{n}} \times \nabla \times \mathbf{P})p\right] \mathrm{d}\mathbf{r} \tag{S28}$$

where p and $\mathbf{P}$ denote continuously differentiable scalar and vector functions, respectively, in the volume $\mathbf{\Omega}$ with a boundary $\partial\mathbf{\Omega}$. $\hat{\mathbf{n}}$ is the unit normal vector on the surface $\partial\mathbf{\Omega}$.

The Huygens's surface (S) is defined between the coil location and the head boundary (as shown in Fig. S1). According to the surface equivalence principle, the impressed currents inside the head can be replaced by equivalent surface electric and magnetic currents on S (Fig. 2B and 2C). In our scenario, we want the fields generated by these surface currents outside the head. As such, we choose $\mathbf{\Omega} \in \mathbb{R}^3/Head$ (i.e., all space without the head). For all $\mathbf{r} \in \mathbb{R}^3/Head$, there are

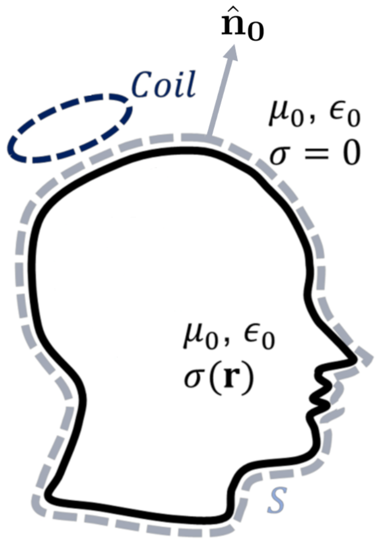

Figure S1: The Huygens's surface (S) is defined between the coil location and the head boundary.

no current sources and no conductive medium. Therefore,

$$\begin{aligned} \nabla \times \mathbf{H_M}(\mathbf{r};t) &= 0, \\ \nabla \times \mathbf{E_M}(\mathbf{r};t) &= -\mu_0 \frac{\mathrm{d}}{\mathrm{d}t}\mathbf{H_M}(\mathbf{r};t), \\ \nabla \times \nabla \times \mathbf{E_M}(\mathbf{r};t) &= 0, \\ \nabla \cdot \epsilon_0 \mathbf{E_M}(\mathbf{r};t) &= 0, \end{aligned} \tag{S29}$$

where $\mathbf{E_M}(\mathbf{r};t)$ and $\mathbf{H_M}(\mathbf{r};t)$ are the E-field and the H-field, respectively, generated by the electric current density sources ($\mathbf{M}(\mathbf{r};t)$), μ_0 is the magnetic permeability in free space, ϵ_0 is the electric permittivity in free space. Since there are no charges, the current continuity follows as $\nabla \cdot \sigma(\mathbf{r})\mathbf{E_M}(\mathbf{r};t) = 0$, or, $\nabla \cdot \mathbf{E_M}(\mathbf{r};t) = 0$.

We now apply $\mathbf{P} = \mathbf{E_M}(\mathbf{r};t)$, $p = g(\mathbf{r} - \mathbf{r}') = \frac{1}{4\pi|\mathbf{r}-\mathbf{r}'|}$ and $\hat{\mathbf{n}} = -\hat{\mathbf{n}}_0$ to Equation (S28), where $g(\mathbf{r} - \mathbf{r}')$ is the free-space scalar Green's function satisfying

$\nabla \cdot \nabla g(\mathbf{r}-\mathbf{r}') = -\delta(\mathbf{r}-\mathbf{r}')$. Here, $\delta(\mathbf{r})$ is the Dirac delta function. Equation (S28) converts to

$$\int_{\mathbb{R}^3/Head} \Big[(\nabla' \cdot \mathbf{E_M}(\mathbf{r}';t))\nabla' g(\mathbf{r}-\mathbf{r}') + \mathbf{E_M}(\mathbf{r}';t)\nabla' \cdot \nabla' g(\mathbf{r}-\mathbf{r}') + g(\mathbf{r}-\mathbf{r}')\nabla' \times \nabla' \times \mathbf{E_M}(\mathbf{r}';t)\Big] \mathrm{d}\mathbf{r}' =$$
$$-\int_{\partial Head} \Big[(\hat{\mathbf{n}}_\mathbf{0}(\mathbf{r}') \cdot \mathbf{E_M}(\mathbf{r}';t))\nabla' g(\mathbf{r}-\mathbf{r}') + \big(\hat{\mathbf{n}}_\mathbf{0}(\mathbf{r}') \times \mathbf{E_M}(\mathbf{r}';t)\big) \times \nabla' g(\mathbf{r}-\mathbf{r}')$$
$$+ (\hat{\mathbf{n}}_\mathbf{0}(\mathbf{r}') \times \nabla' \times \mathbf{E_M}(\mathbf{r}';t))g(\mathbf{r}-\mathbf{r}')\Big] \mathrm{d}\mathbf{r}'. \tag{S30}$$

Using the expressions in Equation (S29), the left-hand side of Equation (S30) simplifies to

$$-\int_{\mathbb{R}^3/Head} \mathbf{E_M}(\mathbf{r}';t)\delta(\mathbf{r}-\mathbf{r}')\mathrm{d}\mathbf{r}' = \begin{cases} -\mathbf{E_M}(\mathbf{r};t), & \text{if } \mathbf{r} \notin Head \\ 0, & \text{if } \mathbf{r} \in Head \end{cases} \tag{S31}$$

whereas the right-hand side takes the form

$$\nabla \int_{\partial Head} g(\mathbf{r}-\mathbf{r}')(\hat{\mathbf{n}}_\mathbf{0}(\mathbf{r}') \cdot \mathbf{E_M}(\mathbf{r}';t))\mathrm{d}\mathbf{r}' - \nabla \times \int_{\partial Head} g(\mathbf{r}-\mathbf{r}')(\hat{\mathbf{n}}_\mathbf{0}(\mathbf{r}') \times \mathbf{E_M}(\mathbf{r}';t))\mathrm{d}\mathbf{r}'$$
$$+ \mu_0 \frac{\mathrm{d}}{\mathrm{d}t} \int_{\partial Head} g(\mathbf{r}-\mathbf{r}')(\hat{\mathbf{n}}_\mathbf{0}(\mathbf{r}') \times \mathbf{H_M}(\mathbf{r}';t))\mathrm{d}\mathbf{r}'. \tag{S32}$$

Now we define the following quantities,

$$\phi(\mathbf{r};t) = \int_{\partial Head} g(\mathbf{r}-\mathbf{r}')(\hat{\mathbf{n}}_\mathbf{0}(\mathbf{r}') \cdot \mathbf{E_M}(\mathbf{r}';t))\mathrm{d}\mathbf{r}' = -\frac{1}{4\pi\epsilon_0}\int_{\partial Head} \frac{\rho_S(\mathbf{r}';t)}{|\mathbf{r}-\mathbf{r}'|}\mathrm{d}\mathbf{r}',$$
$$\mathbf{F}(\mathbf{r};t) = -\int_{\partial Head} g(\mathbf{r}-\mathbf{r}')(\hat{\mathbf{n}}_\mathbf{0}(\mathbf{r}') \times \mathbf{E_M}(\mathbf{r}';t))\mathrm{d}\mathbf{r}' = \frac{1}{4\pi}\int_{\partial Head} \frac{\mathbf{K_S}(\mathbf{r}';t)}{|\mathbf{r}-\mathbf{r}'|}\mathrm{d}\mathbf{r}',$$
$$\mathbf{A}(\mathbf{r};t) = \int_{\partial Head} g(\mathbf{r}-\mathbf{r}')(\hat{\mathbf{n}}_\mathbf{0}(\mathbf{r}') \times \mathbf{H_M}(\mathbf{r}';t))\mathrm{d}\mathbf{r}' = \frac{1}{4\pi}\int_{\partial Head} \frac{\mathbf{J_S}(\mathbf{r}';t)}{|\mathbf{r}-\mathbf{r}'|}\mathrm{d}\mathbf{r}', \tag{S33}$$

where

$$\rho_S(\mathbf{r};t) = -\hat{\mathbf{n}}_\mathbf{0}(\mathbf{r}) \cdot \epsilon_0 \mathbf{E_M}(\mathbf{r};t),$$
$$\mathbf{K_S}(\mathbf{r};t) = -\hat{\mathbf{n}}_\mathbf{0}(\mathbf{r}) \times \mathbf{E_M}(\mathbf{r};t),$$
$$\mathbf{J_S}(\mathbf{r};t) = \hat{\mathbf{n}}_\mathbf{0}(\mathbf{r}) \times \mathbf{H_M}(\mathbf{r};t).$$

Here, $\phi(\mathbf{r};t)$ is the scalar potential, $\rho_S(\mathbf{r};t)$ is the charge density on S, $\mathbf{J_S}(\mathbf{r};t)$ is the electric current density on S, $\mathbf{K_S}(\mathbf{r};t)$ is the magnetic current density on S, $\mathbf{F}(\mathbf{r};t)$ is the magnetic vector potential and $\mathbf{A}(\mathbf{r};t)$ is the electric vector potential. Denoting the E-field outside the head as $\mathbf{E_{J_SK_S}}(\mathbf{r};t)$ generated by $\mathbf{J_S}(\mathbf{r};t)$ and $\mathbf{K_S}(\mathbf{r};t)$, the E-field outside the head takes the form

$$\mathbf{E_{J_SK_S}}(\mathbf{r};t) = \mathbf{E_M}(\mathbf{r};t) = -\nabla\phi(\mathbf{r};t) - \nabla\times\mathbf{F}(\mathbf{r};t) - \mu_0\frac{\mathrm{d}}{\mathrm{d}t}\mathbf{A}(\mathbf{r};t). \tag{S34}$$

Following the discussion in section (6.2), we apply the quasi-static assumptions resulting in $\mathbf{J_S}(\mathbf{r};t) = \hat{\mathbf{n}}_\mathbf{0}(\mathbf{r})\times \mathrm{I}(t)\mathbf{H_M}(\mathbf{r})$ and $\mathbf{K_S}(\mathbf{r};t) = -\hat{\mathbf{n}}_\mathbf{0}(\mathbf{r})\times \mathrm{I}'(t)\mathbf{E_M}(\mathbf{r})$ which can be directly related to Equations (14).

6.5. Proof that surface equivalent currents are radiating in free space

The equivalent surface electric current density ($\mathbf{J_S}(\mathbf{r};t)$) and magnetic current density ($\mathbf{K_S}(\mathbf{r};t)$) generate E-field ($\mathbf{E_{J_S,K_S}}(\mathbf{r};t)$) and H-field ($\mathbf{H_{J_S,K_S}}(\mathbf{r};t)$) outside the Huygens's surface (S) which follow the Maxwell's Equations as

$$\begin{aligned}
&\nabla\times\mathbf{H_{J_S,K_S}}(\mathbf{r};t) = \mathbf{J_S}(\mathbf{r};t),\\
&\nabla\times\mathbf{E_{J_S,K_S}}(\mathbf{r};t) = -\mu_0\frac{\mathrm{d}}{\mathrm{d}t}\mathbf{H_{J_S,K_S}}(\mathbf{r};t) - \mathbf{K_S}(\mathbf{r};t),\\
&\nabla\cdot\epsilon_0\mathbf{E_{J_S,K_S}}(\mathbf{r};t) = \rho_S(\mathbf{r};t),
\end{aligned} \tag{S35}$$

where ρ_S is the charge density on the surface S. Now assume the E-field is represented as shown in Equation (S34),

$$\begin{aligned}
&\mathbf{E_{J_S,K_S}}(\mathbf{r};t) = -\nabla\phi_{J_S,K_S}(\mathbf{r};t) - \nabla\times\mathbf{F_{J_S,K_S}}(\mathbf{r};t) - \mu_0\frac{\mathrm{d}}{\mathrm{d}t}\mathbf{A_{J_S,K_S}}(\mathbf{r};t),\\
&\mathbf{H_{J_S,K_S}}(\mathbf{r};t) = \nabla\times\mathbf{A_{J_S,K_S}}(\mathbf{r};t),\\
&\nabla\cdot\mathbf{A_{J_S,K_S}}(\mathbf{r};t) = 0,\\
&\nabla\cdot\mathbf{F_{J_S,K_S}}(\mathbf{r};t) = 0,
\end{aligned} \tag{S36}$$

where $\mathbf{A}_{\mathbf{J_S},\mathbf{K_S}}(\mathbf{r};t)$ is the electric vector potential, $\mathbf{F}_{\mathbf{J_S},\mathbf{K_S}}(\mathbf{r};t)$ is the magnetic vector potential and $\phi_{J_S,K_S}(\mathbf{r};t)$ is the scalar potential generated by electric current density ($\mathbf{J_S}(\mathbf{r};t)$) and magnetic current density ($\mathbf{K_S}(\mathbf{r};t)$). Now, taking the curl of the H-field results in

$$\nabla \times \mathbf{H}_{\mathbf{J_S},\mathbf{K_S}}(\mathbf{r};t) = \nabla(\nabla \cdot \mathbf{A}_{\mathbf{J_S},\mathbf{K_S}}(\mathbf{r};t)) - \nabla^2 \mathbf{A}_{\mathbf{J_S},\mathbf{K_S}}(\mathbf{r};t) = \mathbf{J_S}(\mathbf{r};t), \tag{S37}$$

or,

$$-\nabla^2 \mathbf{A}_{\mathbf{J_S},\mathbf{K_S}}(\mathbf{r};t) = \mathbf{J_S}(\mathbf{r};t). \tag{S38}$$

Using a Green's function, the electric vector potential can be solved as

$$\mathbf{A}_{\mathbf{J_S},\mathbf{K_S}}(\mathbf{r};t) = \frac{1}{4\pi}\int_S \frac{\mathbf{J_S}(\mathbf{r}';t)}{|\mathbf{r}-\mathbf{r}'|}\mathrm{d}\mathbf{r}'. \tag{S39}$$

Taking the curl of the E-field results in

$$\begin{aligned} \nabla \times \mathbf{E}_{\mathbf{J_S},\mathbf{K_S}}(\mathbf{r};t) &= \nabla \times \left[-\nabla \phi_{J_S,K_S}(\mathbf{r};t) - \nabla \times \mathbf{F}_{\mathbf{J_S},\mathbf{K_S}}(\mathbf{r};t) - \mu_0 \frac{\mathrm{d}}{\mathrm{d}t}\mathbf{A}_{\mathbf{J_S},\mathbf{K_S}}(\mathbf{r};t) \right], \\ &= -\mu_0 \frac{\mathrm{d}}{\mathrm{d}t}\mathbf{H}_{\mathbf{J_S},\mathbf{K_S}}(\mathbf{r};t) - \mathbf{K_S}(\mathbf{r};t), \end{aligned} \tag{S40}$$

or,

$$\nabla \times \nabla \times \mathbf{F}_{\mathbf{J_S},\mathbf{K_S}}(\mathbf{r};t) = -\nabla^2 \mathbf{F}_{\mathbf{J_S},\mathbf{K_S}}(\mathbf{r};t) = \mathbf{K_S}(\mathbf{r};t). \tag{S41}$$

We have assumed $\nabla \cdot \mathbf{F} = 0$. Using a Green's function,

$$\mathbf{F}_{\mathbf{J_S},\mathbf{K_S}}(\mathbf{r};t) = \frac{1}{4\pi}\int_S \frac{\mathbf{K_S}(\mathbf{r}';t)}{|\mathbf{r}-\mathbf{r}'|}\mathrm{d}\mathbf{r}'. \tag{S42}$$

Finally, taking the divergence of the E-field results in

$$\nabla \cdot \epsilon_0 \mathbf{E}_{\mathbf{J_S},\mathbf{K_S}}(\mathbf{r};t) = \nabla \cdot \epsilon_0 \left[-\nabla \phi_{J_S,K_S}(\mathbf{r};t) - \nabla \times \mathbf{F}_{\mathbf{J_S},\mathbf{K_S}}(\mathbf{r};t) - \mu_0 \frac{\mathrm{d}}{\mathrm{d}t} \mathbf{A}_{\mathbf{J_S},\mathbf{K_S}}(\mathbf{r};t) \right] = \rho_S, \tag{S43}$$

or,

$$-\epsilon_0 \nabla^2 \phi_{J_S,K_S}(\mathbf{r};t) = \rho_S. \tag{S44}$$

Using the Green's function,

$$\phi_{J_S,K_S}(\mathbf{r};t) = \frac{1}{4\pi\epsilon_0} \int_S \frac{\rho_\mathbf{S}(\mathbf{r}';t)}{|\mathbf{r}-\mathbf{r}'|} \mathrm{d}\mathbf{r}'. \tag{S45}$$

Equations (S39), (S42) and S45 show the $\mathbf{A}_{\mathbf{J_S},\mathbf{K_S}}(\mathbf{r};t)$, $\mathbf{F}_{\mathbf{J_S},\mathbf{K_S}}(\mathbf{r};t)$ and $\phi_{J_S,K_S}(\mathbf{r};t)$ in free space due to $\mathbf{J_S}(\mathbf{r};t)$, $\mathbf{K_S}(\mathbf{r};t)$ and $\rho_S(\mathbf{r};t)$, respectively.

6.6. Reciprocity principle using equivalent currents on Huygens's surface

In this section, we form the reciprocity relation between the fields generated by equivalent surface currents on Huygens's surface and the primary fields generated by the coil. In the first scenario, the equivalent surface electric current density ($\mathbf{J_S}(\mathbf{r};t)$) and magnetic current density ($\mathbf{K_S}(\mathbf{r};t)$) generate E-field ($\mathbf{E}_{\mathbf{J_S},\mathbf{K_S}}(\mathbf{r};t)$) and H-field ($\mathbf{H}_{\mathbf{J_S},\mathbf{K_S}}(\mathbf{r};t)$). According to Faraday's and Ampere's law,

$$\nabla \times \mathbf{E}_{\mathbf{J_S},\mathbf{K_S}}(\mathbf{r};t) = -\mu_0 \frac{\mathrm{d}}{\mathrm{d}t} \mathbf{H}_{\mathbf{J_S},\mathbf{K_S}}(\mathbf{r};t) - \mathbf{K_S}(\mathbf{r};t), \tag{S46a}$$

$$\nabla \times \mathbf{H}_{\mathbf{J_S},\mathbf{K_S}}(\mathbf{r};t) = \mathbf{J_S}(\mathbf{r};t), \tag{S46b}$$

respectively. In the second scenario, the primary E-field ($\mathbf{E}^{\mathbf{P}}_{\mathbf{TMS}}(\mathbf{r};t)$) and H-field ($\mathbf{H}^{\mathbf{P}}_{\mathbf{TMS}}(\mathbf{r};t)$) generated by the coil current density ($\mathbf{J}_{\mathbf{TMS}}(\mathbf{r};t)$) satisfy the following equations,

$$\nabla \times \mathbf{E}^{\mathbf{P}}_{\mathbf{TMS}}(\mathbf{r};t) = -\mu_0 \frac{\mathrm{d}}{\mathrm{d}t}\mathbf{H}^{\mathbf{P}}_{\mathbf{TMS}}(\mathbf{r};t), \tag{S47a}$$

$$\nabla \times \mathbf{H}^{\mathbf{P}}_{\mathbf{TMS}}(\mathbf{r};t) = \mathbf{J}_{\mathbf{TMS}}(\mathbf{r};t). \tag{S47b}$$

In both cases, the displacement currents are neglected. Now, Equation (S47b) dotted with $\mathbf{E}_{\mathbf{J_S},\mathbf{K_S}}(\mathbf{r};t)$ is subtracted from Equation (S46a) dotted with $\mathbf{H}^{\mathbf{P}}_{\mathbf{TMS}}(\mathbf{r};t)$ resulting in

$$\begin{aligned}\mathbf{H}^{\mathbf{P}}_{\mathbf{TMS}}(\mathbf{r};t)\cdot\nabla\times\mathbf{E}_{\mathbf{J_S},\mathbf{K_S}}(\mathbf{r};t) - \mathbf{E}_{\mathbf{J_S},\mathbf{K_S}}(\mathbf{r};t)\cdot\nabla\times\mathbf{H}^{\mathbf{P}}_{\mathbf{TMS}}(\mathbf{r};t) &= \nabla\cdot(\mathbf{E}_{\mathbf{J_S},\mathbf{K_S}}(\mathbf{r};t)\times\mathbf{H}^{\mathbf{P}}_{\mathbf{TMS}}(\mathbf{r};t))\\ = -\mu_0\frac{\mathrm{d}}{\mathrm{d}t}\mathbf{H}^{\mathbf{P}}_{\mathbf{TMS}}(\mathbf{r};t)\cdot\mathbf{H}_{\mathbf{J_S},\mathbf{K_S}}(\mathbf{r};t) - \mathbf{H}^{\mathbf{P}}_{\mathbf{TMS}}(\mathbf{r};t)\cdot\mathbf{K_S}(\mathbf{r};t) &- \mathbf{E}_{\mathbf{J_S},\mathbf{K_S}}(\mathbf{r};t)\cdot\mathbf{J}_{\mathbf{TMS}}(\mathbf{r};t).\end{aligned} \tag{S48}$$

Using the vector identity $\nabla\cdot(\mathbf{A}\times\mathbf{B}) = \mathbf{B}\cdot(\nabla\times\mathbf{A}) - \mathbf{A}\cdot(\nabla\times\mathbf{B})$, the left-hand-side of Equation (S48) becomes $\nabla\cdot(\mathbf{E}_{\mathbf{J_S},\mathbf{K_S}}(\mathbf{r};t)\times\mathbf{H}^{\mathbf{P}}_{\mathbf{TMS}}(\mathbf{r};t))$. In a similar manner, Equation (S46b) dotted with $\mathbf{E}^{\mathbf{P}}_{\mathbf{TMS}}(\mathbf{r};t)$ is subtracted from Equation (S47a) dotted with $\mathbf{H}_{\mathbf{J_S},\mathbf{K_S}}(\mathbf{r};t)$ resulting in

$$\begin{aligned}\mathbf{H}_{\mathbf{J_S},\mathbf{K_S}}(\mathbf{r};t)\cdot\nabla\times\mathbf{E}^{\mathbf{P}}_{\mathbf{TMS}}(\mathbf{r};t) - \mathbf{E}^{\mathbf{P}}_{\mathbf{TMS}}(\mathbf{r};t)\cdot\nabla\times\mathbf{H}_{\mathbf{J_S},\mathbf{K_S}}(\mathbf{r};t) &= \nabla\cdot(\mathbf{E}^{\mathbf{P}}_{\mathbf{TMS}}(\mathbf{r};t)\times\mathbf{H}_{\mathbf{J_S},\mathbf{K_S}}(\mathbf{r};t))\\ = -\mu_0\frac{\mathrm{d}}{\mathrm{d}t}\mathbf{H}_{\mathbf{J_S},\mathbf{K_S}}(\mathbf{r};t)\cdot\mathbf{H}^{\mathbf{P}}_{\mathbf{TMS}}(\mathbf{r};t) - \mathbf{E}^{\mathbf{P}}_{\mathbf{TMS}}(\mathbf{r};t)\cdot\mathbf{J_S}(\mathbf{r};t).&\end{aligned} \tag{S49}$$

Next, by subtracting Equation (S48) from (S49), we get

$$\begin{aligned}\nabla\cdot\left[\mathbf{E}^{\mathbf{P}}_{\mathbf{TMS}}(\mathbf{r};t)\times\mathbf{H}_{\mathbf{J_S},\mathbf{K_S}}(\mathbf{r};t) - \mathbf{E}_{\mathbf{J_S},\mathbf{K_S}}(\mathbf{r};t)\times\mathbf{H}^{\mathbf{P}}_{\mathbf{TMS}}(\mathbf{r};t)\right] &=\\ -\mathbf{E}^{\mathbf{P}}_{\mathbf{TMS}}(\mathbf{r};t)\cdot\mathbf{J_S}(\mathbf{r};t) + \mathbf{H}^{\mathbf{P}}_{\mathbf{TMS}}(\mathbf{r};t)\cdot\mathbf{K_S}(\mathbf{r};t) &+ \mathbf{E}_{\mathbf{J_S},\mathbf{K_S}}(\mathbf{r};t)\cdot\mathbf{J}_{\mathbf{TMS}}(\mathbf{r};t).\end{aligned} \tag{S50}$$

Integrating Equation (S50) over the volume $\mathbf{\Omega}$ with the boundary $\partial\mathbf{\Omega}$ and applying Gauss's law, we have

$$\int_{\partial\Omega}\left[\mathbf{E}^{\mathbf{P}}_{\mathbf{TMS}}(\mathbf{r};t)\times\mathbf{H}_{\mathbf{J_S},\mathbf{K_S}}(\mathbf{r};t)-\mathbf{E}_{\mathbf{J_S},\mathbf{K_S}}(\mathbf{r};t)\times\mathbf{H}^{\mathbf{P}}_{\mathbf{TMS}}(\mathbf{r};t)\right]\cdot d\mathbf{r} = -\int_{\Omega}\mathbf{E}^{\mathbf{P}}_{\mathbf{TMS}}(\mathbf{r};t)\cdot\mathbf{J_S}(\mathbf{r};t)d\mathbf{r}+\int_{\Omega}\mathbf{H}^{\mathbf{P}}_{\mathbf{TMS}}(\mathbf{r};t)\cdot\mathbf{K_S}(\mathbf{r};t)d\mathbf{r}+\int_{\Omega}\mathbf{E}_{\mathbf{J_S},\mathbf{K_S}}(\mathbf{r};t)\cdot\mathbf{J_{TMS}}(\mathbf{r};t)d\mathbf{r}. \tag{S51}$$

Let's assume the sources $\mathbf{J_S}(\mathbf{r};t)$ and $\mathbf{K_S}(\mathbf{r};t)$ can be enclosed by finite-sized small circular elements on the surface S. Then for an infinite-sized sphere, $\mathbf{H}_{\mathbf{J_S},\mathbf{K_S}}(\mathbf{r};t)=\hat{\mathbf{r}}\times\frac{\mathbf{E}_{\mathbf{J_S},\mathbf{K_S}}(\mathbf{r};t)}{\eta}$, $\mathbf{H}^{\mathbf{P}}_{\mathbf{TMS}}(\mathbf{r};t)=\hat{\mathbf{r}}\times\frac{\mathbf{E}^{\mathbf{P}}_{\mathbf{TMS}}(\mathbf{r};t)}{\eta}$, $\mathbf{E}_{\mathbf{J_S},\mathbf{K_S}}(\mathbf{r};t)\cdot\hat{\mathbf{r}}=\mathbf{H}_{\mathbf{J_S},\mathbf{K_S}}(\mathbf{r};t)\cdot\hat{\mathbf{r}}=\mathbf{H}^{\mathbf{P}}_{\mathbf{TMS}}(\mathbf{r};t)\cdot\hat{\mathbf{r}}=\mathbf{E}^{\mathbf{P}}_{\mathbf{TMS}}(\mathbf{r};t)\cdot\hat{\mathbf{r}}=0$. Here, η is the free-space impedance (Kong, 1986). With these source definitions and the vector identity $\mathbf{A}\times(\mathbf{B}\times\mathbf{C})=\mathbf{B}(\mathbf{A}\cdot\mathbf{C})-\mathbf{C}(\mathbf{A}\cdot\mathbf{B})$, the left-hand-side of Equation (S51) is evaluated to zero. Equation (S51) takes the form

$$\int_{\Omega}\mathbf{E}_{\mathbf{J_S},\mathbf{K_S}}(\mathbf{r};t)\cdot\mathbf{J_{TMS}}(\mathbf{r};t)d\mathbf{r}=\int_{\Omega}\mathbf{E}^{\mathbf{P}}_{\mathbf{TMS}}(\mathbf{r};t)\cdot\mathbf{J_S}(\mathbf{r};t)d\mathbf{r}-\int_{\Omega}\mathbf{H}^{\mathbf{P}}_{\mathbf{TMS}}(\mathbf{r};t)\cdot\mathbf{K_S}(\mathbf{r};t)d\mathbf{r}. \tag{S52}$$

Using the quasi-static assumption and since $\mathbf{J_S}(\mathbf{r};t)$ and $\mathbf{K_S}(\mathbf{r};t)$ only reside on the surface (S) and $\mathbf{J_{TMS}}(\mathbf{r};t)$ is only defined at coil location, Equation (S52) takes the final form

$$\begin{aligned}\int_{Coil}\mathrm{I}'(t)\mathbf{E}_{\mathbf{J_S},\mathbf{K_S}}(\mathbf{r})\cdot\mathrm{I}(t)\mathbf{J_{TMS}}(\mathbf{r})d\mathbf{r} &= \int_{S}\left[\mathrm{I}'(t)\mathbf{E}^{\mathbf{P}}_{\mathbf{TMS}}(\mathbf{r})\cdot\mathrm{I}(t)\mathbf{J_S}(\mathbf{r})d\mathbf{r}-\mathrm{I}(t)\mathbf{H}^{\mathbf{P}}_{\mathbf{TMS}}(\mathbf{r};t)\cdot\mathrm{I}'(t)\mathbf{K_S}(\mathbf{r})\right]d\mathbf{r},\\ &= \mathrm{I}(t)\mathrm{I}'(t)\int_{S}\left[\mathbf{E}^{\mathbf{P}}_{\mathbf{TMS}}(\mathbf{r})\cdot\mathbf{J_S}(\mathbf{r})d\mathbf{r}-\mathbf{H}^{\mathbf{P}}_{\mathbf{TMS}}(\mathbf{r};t)\cdot\mathbf{K_S}(\mathbf{r})\right]d\mathbf{r}.\end{aligned} \tag{S53}$$

The reciprocity form in Equation (S53) can be directly related to Equation (10).

6.7. FLOPS and memory bandwidth at real-time stage

Here we estimate the number of floating point operations in the GPU during the real-time stage. First, we must compute the inverse of a 4 by 4 coordinate transformation matrix, which,

assuming computational cost $23n^3$, where n is the size of the matrix, requires 1472 FLOPS. For each of Huygens's surface points, we must apply the inverse coordinate transform which involves N_d matrix vector multiplies with the 4 by 4 inverse coordinate transform matrix. This requires $2 \cdot 4 \cdot 4 \cdot N_d = 32N_d$ operations.

The tri-linear interpolation to find the primary E-field is more complicated. For each of the N_d locations, we must find its grid box and relative distance from the grid points of it. This is done by taking each coordinate and dividing it by the vertex grid spacing. We must take each coordinate: (1) divide it by the grid spacing, (2) compute the box index as the floor of the result (3) the remainder is the relative distance from the vertices that are nearest to the coordinate axis, (4) one minus the reminder relative distance from the further ones. We assume each of these steps requires one FLOP and there are three coordinates per location on the Huygens' surface. As such the total cost is assumed to be $4 \cdot 3 \cdot N_d = 12N_d$. The multi-linear interpolation requires the multiplication of the relative distances along each of the coordinate axes with the function value at its corresponding vertex (i.e. 4 multiplications per vertex). There are 8 vertices and the results from each vertex must be added to determine the interpolation value. As such, each interpolation requires $4 \cdot 8 + 8 = 40$ FLOPS. An interpolation has to be done for each of the Cartesian coordinate components of both $\mathbf{E}^{\mathbf{P}}_{\mathbf{TMS}}(\mathbf{r})$ and $\mathbf{H}^{\mathbf{P}}_{\mathbf{TMS}}(\mathbf{r})$. As such, the total FLOPS for the interpolation are $40 \cdot 6N_d + 12N_d = 252N_d$.

The primary E-fields must be rotated to be in the same coordinate reference system as the Huygens's currents. This is multiplication with each of the interpolated E-fields and H-fields by a 3 by 3 rotation matrix, which requires a total of $2 \cdot 3 \cdot 3 \cdot (2N_d) = 36N_d$. Next, evaluation of Equation (11) (i.e. coefficients) requires a matrix-vector multiplication with a matrix that is N_m by $6N_d$, which requires $2 \cdot N_m \cdot 6N_d = 12N_mN_d$ FLOPS. Finally, determining the E-fields at N_e locations requires a matrix-vector multiplication with a matrix that is$3N_e$ by N_m, which requires $2 \cdot 3N_e \cdot N_m = 6N_eN_m$ FLOPS. In total the approximate FLOPS required are $1472 + (32 + 252 + 36)N_d + 12N_mN_d + 6N_mN_e$. In this paper, on average, $N_d = 120,000$, $N_m = 400$ and $N_e = 250,000$. Therefore, the FLOP count is around 1.2×10^9. Note these are only estimates provided here for the interested reader on the actual computation being done during the

real-time step. Better estimates can be obtained by actually using compiler information about how each operation is implemented in hardware. For example, per the theoretical values of the NVIDIA GPU here the number of theoretical single precision operations per second is 29.77 TFLOPS. Using this as an estimate for the total computation it predicts a computation time of 0.038 ms, which is much lower than what we observe.

The important details of this analysis are that the computation increases as the product of modes and the number of Huygens' surface points plus the product of the number of modes and the number of brain E-field evaluation points. As such, for fixed accuracy, the computation time increases linearly with the number of samples.

As per the memory requirements, we need to store $6N_dN_m$ numbers for $\mathbf{J}_S(\mathbf{r})$ and $\mathbf{K}_S(\mathbf{r})$, $9N_g^3$ numbers for the primary E-fields, H-fields, and grid node Cartesian locations, $12N_d$ numbers for the Huygens' surface $\mathbf{E}^{\mathbf{P}}_{\mathbf{TMS}}(\mathbf{r})$, $\mathbf{H}^{\mathbf{P}}_{\mathbf{TMS}}(\mathbf{r})$, Cartesian locations, and transformed Cartesian locations, N_m numbers for the expansion coefficients, and $3N_eN_m$ numbers for the matrix that takes expansion coefficients to brain E-fields, $3N_e$ numbers for brain E-field samples, and $16 \cdot 2$ numbers for the coordinate transformation matrices. As such the total numbers that need to be stored is $6N_d(N_m+2)+3N_e(N_m+1)+9N_g^3+N_m+32$ at 4 bytes per single number and using $N_g^3 = 822016$ a typical calculation should require 2.2 GBs plus standard overhead of GPU memory, which is close to the 3.0 GBs that was observed.

6.8. Effect of interpolation grid density

Fig. (S2) shows the relative error (GME) with respect to the interpolation grid density for 1000 random Coil placements over the scalp of a subject when the number of modes is fixed at 400. There is negligible change in the performance until a grid spacing of 5 mm in the order of 10^{-6}. On the other hand, at extremely low grid dimensions, the required memory increases which leaves less room for the modes to be accommodated in the GPU memory.

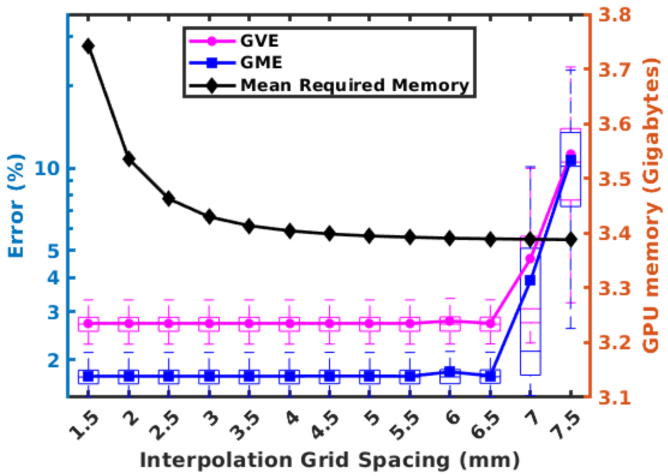

Figure S2: Effect of interpolation grid density on real-time TMS E-field prediction. The average error remains almost constant until a grid spacing of 5 mm. On the right axis, the effect on GPU memory is shown.

For lower grid dimensions (denser grid), the required memory increases rapidly.

6.9. Effect of FEM order

Fig. (S3) shows the effect of FEM order on the convergence of error. For higher FEM orders, the convergence is slower in both GME and GVE. For example, we conducted this study on the 'Ernie' head model from SimNIBS (Thielscher et al., 2015). For first-order FEM, the GME and GVE reach the 2% error bound at 360 and 480, respectively. On the other hand, for second-order FEM, the same error bound is reached at 290 and 330, respectively. But, the trade-off is the time and memory requirement in the offline stage. The real-time stage is completely unaffected by the order of the FEM. However, if the targeted error limit is very high, the improvement becomes negligible.

6.10. Accuracy of E-field due to real-time TMS and 1st-order FEM

Here, we show the relative accuracy of the real-time predicted TMS E-field and the 1st-order FEM with respect to the 2nd-order FEM. We compute error estimates for both the real-time (GVE_{RT} and GME_{RT}) and 1st-order FEM (GVE_{FEM} and GME_{FEM}) relative to a 2nd-order FEM reference solution.Fig. (S4) shows the convergence of the average errors with respect to modes. Fig. (S5) shows the same differences between the GVE_{RT} and the GVE_{FEM} for each mode across 16000

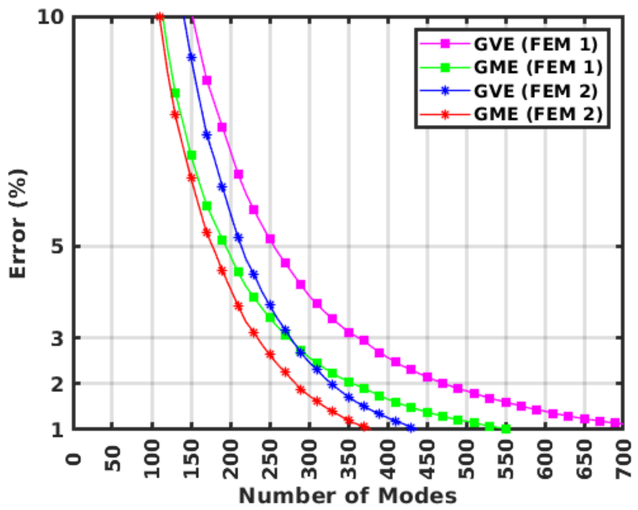

Figure S3: Effect of FEM on the error of real-time TMS E-field prediction. Higher order FEM facilitates less number of modes for a targeted error bound.

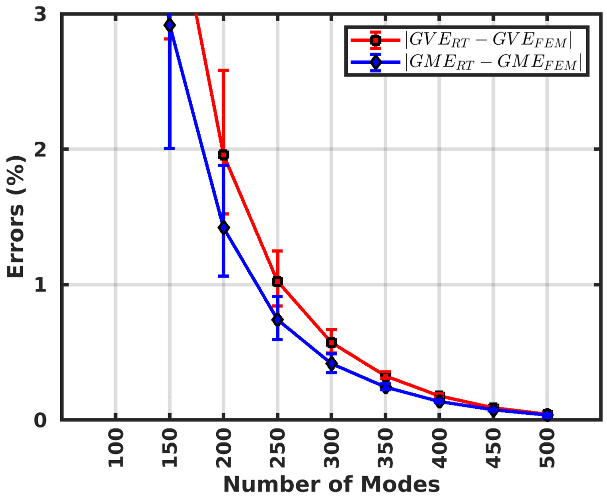

Figure S4: Error (GVE and GME) comparison between real-time TMS E-field and 1st-order FEM-induced E-field with respect to the 2nd-order FEM-induced E-field. The distribution at any rank (mode) is across 16000 random simulations (1000 random Coil placements over the scalp of each of 16 head models).

simulations. A positive value of difference indicates that for that particular solution, the 1st-order FEM is more accurate than the real-time solution, and a negative difference indicates that the real-time solution is more accurate. We observe that for 250 modes, the 1st-order FEM is on average 1.0 % more accurate than the real-time, and for 400 modes, on average, the real-time is just as accurate as the 1st-order FEM. Furthermore, for 400 modes, in the worst-case scenario, the real-time is

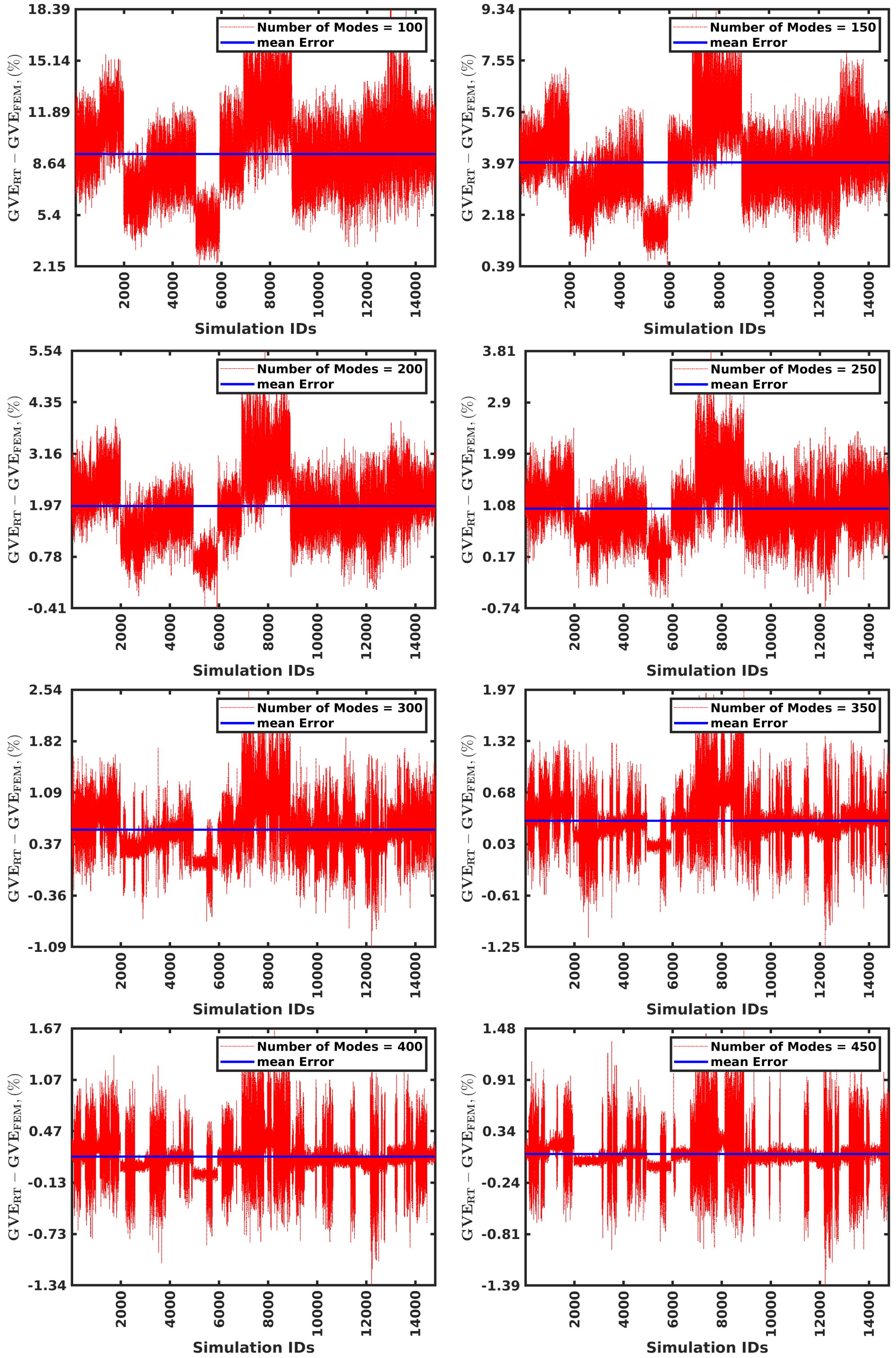

Figure S5: Error (GVE) comparison between real-time TMS E-field and 1st-order FEM-induced E-field with respect to the 2nd-order FEM-induced E-field across 16000 simulations for the ranks of 100, 150, 200, 250, 300, 350, 400, and 450.

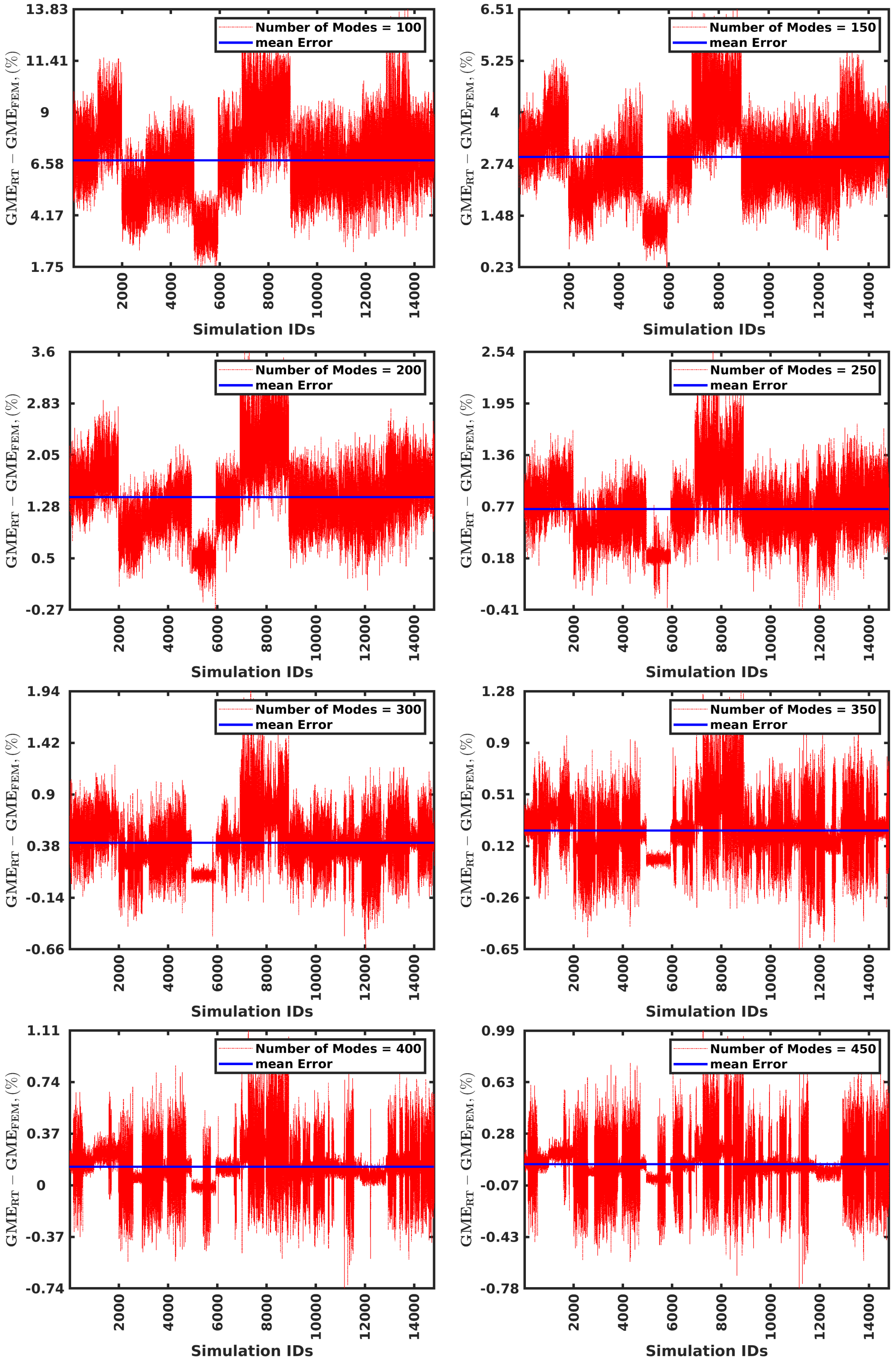

Figure S6: Error (GME) comparison between real-time TMS E-field and 1st-order FEM-induced E-field with respect to the 2nd-order FEM-induced E-field across 16000 simulations for the ranks of 100, 150, 200, 250, 300, 350, 400, and 450.

2.54 % less accurate than 1st-order FEM and in the best-case scenario, 1.09 % more accurate. This indicates that using 400 modes is likely a judicious choice.

For most applications, the magnitude of the E-field is used as the figure of merit. Fig. (S6) shows the difference between the GME_{RT} and the GME_{FEM}. Again, a positive value of difference indicates that for that particular solution the 1st-order FEM is more accurate than the real-time solution and a negative difference indicates the real-time solution is more accurate. We observe that for 250 modes, the 1st-order FEM is on average 0.77 % more accurate than the real-time, and for 400 modes, on average, the real-time is nearly just as accurate as the 1st-order FEM. Furthermore, for 400 modes, in the worst-case scenario, the real-time is 1.67 % less accurate than 1st-order FEM and in the best-case scenario, 1.34 % more accurate.

At rank 400, the real-time predicted E-field almost perfectly matched the 1st-order FEM accuracy. These plots indicate that beyond 400 modes our real-time solver and 1st-order FEM are just as accurate because the two match a more accurate solution just as well.

6.11. Generation of Huygens's surface

The Huygens's surface is generated by extruding the center point of scalp triangular facets normally outward by 1 mm. Fig. (S7) illustrates the process.

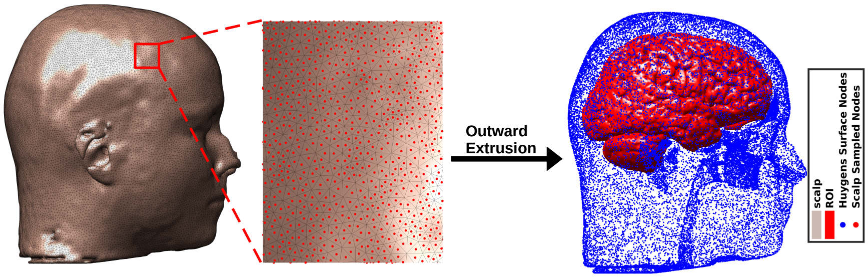

Figure S7: The Huygens's surface generation by extruding scalp sampled points.

6.12. Inverse relative transformation of Huygens's surface

Fig. (S8) shows graphically the inverse relative transformation of the Huygens's surface with respect to the coil. The transformed Huygens's surface nodes may (or not) overlap the grid nodes. Therefore, a multilinear interpolation helps sample the E-field on the Huygens's surface.

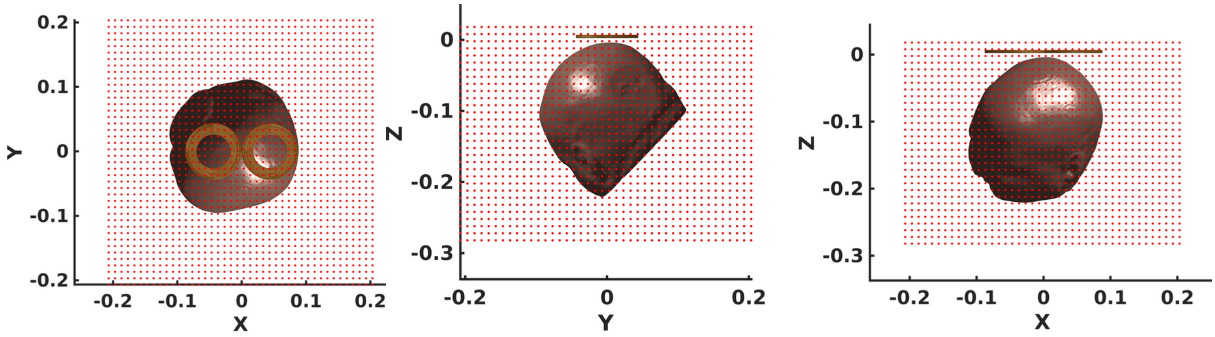

Figure S8: Inverse relative transformation of the Huygens's surface with respect to the coil.

6.13. Error distribution across the Cortex

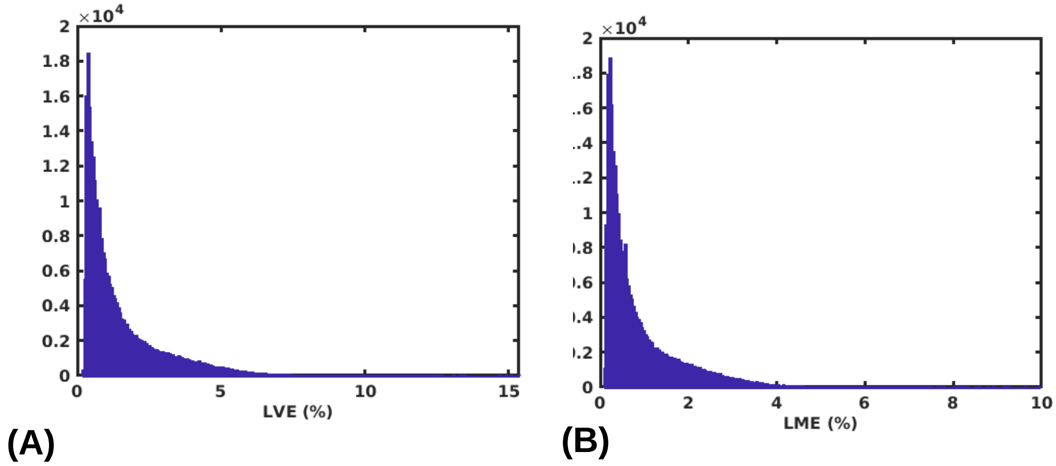

Figure S9: mean LVE and mean LME distribution over the cortex.

Fig. (S9) shows the mean error distribution (mean LVE and mean LME) over the cortex computed across 1000 simulations for a single subject
